# Predicting Metalloprotein Redox Potentials with Machine Learning: A Focus on Iron–Sulfur Systems

**DOI:** 10.1021/acs.jcim.5c01752

**Published:** 2025-10-30

**Authors:** Francesca Persico, Bruno G. Galuzzi, Miriana Pellegrino, Anne-Lise Claudel, Luca De Gioia, Flavia Nastri, Gianfranco Gilardi, Chiara Damiani, Francesca Valetti, Marco Chino, Federica Arrigoni

**Affiliations:** † Department of Biotechnology and Biosciences, University of Milano-Bicocca, Piazza Dell’Ateneo Nuovo 1, Milano 20126, Italy; ‡ Institute of Bioimaging and Complex Biological Systems, 9327 National Research Council, Via Fratelli Cervi 96, Segrate 20054, Italy; § National Biodiversity Future Center, Piazza Marina 61, Palermo 90133, Italy; ∥ Department of Chemical Sciences, University of Naples “Federico II”, Via Cinthia 26, Napoli 80126, Italy; ⊥ Department of Life Sciences and Systems Biology, University of Torino, Via Verdi 8, Torino 10124, Italy; # Laboratorio InfoLife, Consorzio Interuniversitario Nazionale per l’Informatica, Via Ariosto 25, Roma 00185, Italy

## Abstract

Iron–Sulfur (Fe–S) proteins play essential roles in a wide range of biological processes, from energy conversion and respiration to DNA repair and redox signaling, making them highly relevant to both bioenergetics and human health. These proteins mediate electron transfer through finely tuned reduction potentials (RP) defined by their metal cofactors. However, predicting RP from protein structures remains a significant challenge due to the complex electronic nature of Fe–S clusters and their intricate coupling with the surrounding protein environment. This complexity limits our ability to systematically modulate RP, hindering efforts in high-throughput and rational protein design. In this study, we introduce a Machine Learning (ML) framework, FeS-RedPred, for accurate and scalable prediction of RP in Fe–S proteins. We focus on mono- and binuclear clusters, such as rubredoxins and [2Fe–2S] clusters of ferredoxins, Rieske, and mitoNEET-type, which serve as ideal model systems thanks to the availability of abundant structural and electrochemical data. Our approach relies on structure-derived molecular descriptors computed across multiple spatial scales, from local atomic environments to global protein-level features. Using Extreme Gradient Boosting (XGB) models, we achieve a mean absolute error of ∼40 mV, which is competitive with state-of-the-art computational approaches, while also providing a highly efficient compromise between accuracy and computational cost. Beyond predictive accuracy, our model also offers indications about the determinants of RP, enabling a basis for interpretation and potentially guiding protein engineering. This work provides a valuable foundation for understanding the redox behavior of metalloproteins, enabling the high-throughput prediction of redox potentials and informing data-driven design across diverse protein families.

## Graphical abstract

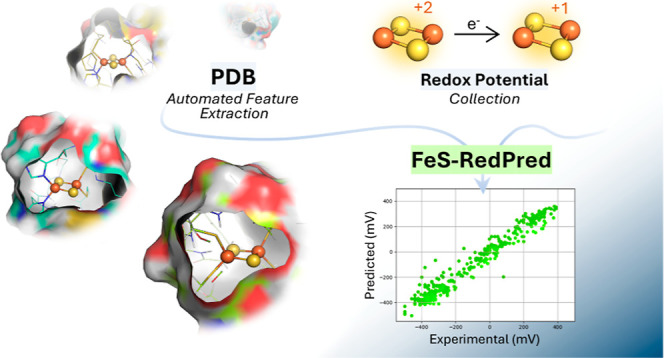

## Introduction

Over billions of years, evolution has shaped enzymes to carry out essential biochemical reactions with remarkable efficiency. Among them, metalloenzymes play a crucial role in electron transfer, mediating redox reactions fundamental to life. To function effectively, they must finely tune their reduction potentials (RP) to align with that of their redox partners. Iron–Sulfur (Fe–S) proteins occupy most of the biological RP spectrum while exhibiting remarkable redox versatility (Figure 1). This diversity reflects their functional adaptability across biological systems, where they serve as highly efficient electron carriers due to the delocalization of electron density over Fe and S atoms. As a result, Fe–S clusters are central to key biological processes such as photosynthesis, respiration, and enzymatic energy conversion in hydrogenases, CO dehydrogenases, formate dehydrogenases, and nitrogenases.^1–4^ Beyond bioenergetics, Fe–S clusters also play roles in cellular sensing, with their redox properties directly linked to DNA repair and the regulation of viral replication.^5–7^

**Figure 1:**
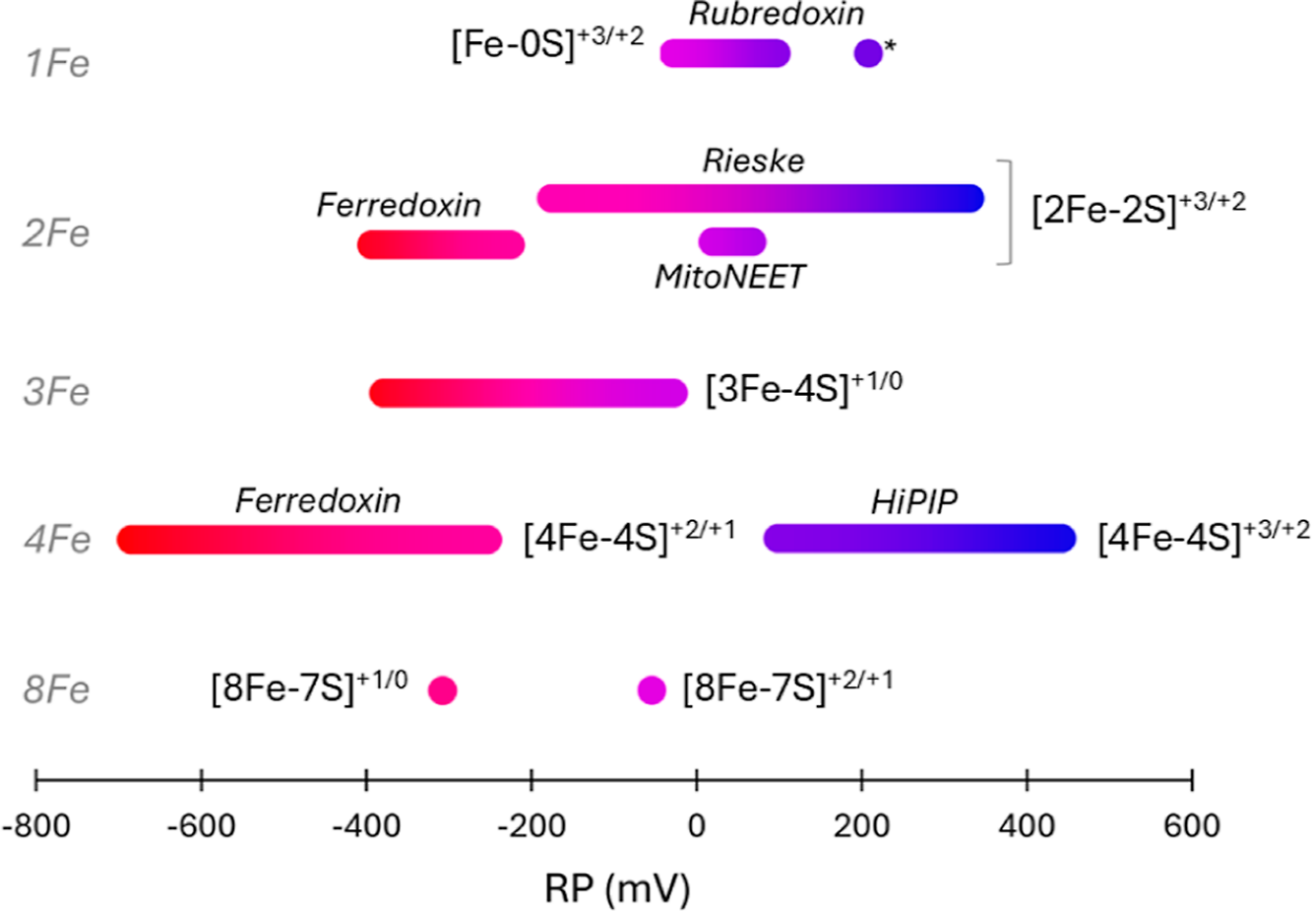
RP distribution of Fe–S clusters in proteins, going from more negative (red) to more positive (blue) values. * Refers to the rubredoxin-type center found in rubrerythrin.^8^ As for [8Fe–7S] clusters, RP values are referred to the first and second reduction of the nitrogenase P-cluster.^9^ [4Fe–4S]-clusters are distinguished between classical ferredoxins and high potential ferredoxins (HiPIPs).

Fe–S clusters exist in various forms, including [Fe–0S], [2Fe–2S], [3Fe–4S], [4Fe–4S], and [8Fe–7S], with iron typically coordinated in a tetrahedral arrangement by (mostly) cysteine residues. Their RP is finely tuned by the surrounding protein environment, optimizing their function for specific biological roles. Understanding how the protein matrix modulates RP is therefore essential not only for unraveling fundamental biochemical principles but also for guiding the rational design of Fe–S proteins with tailored redox properties for biotechnological human-defined applications. Numerous studies have investigated the influence of the protein environment on Fe–S redox properties, employing both experimental and computational approaches.^3,10–26^ Since the early 1990s, density functional theory (DFT)-based or hybrid (QM/MM) methods, well-suited to account for antiferromagnetic coupling, have provided valuable insights into these effects.^27–31^ In alternative approaches, RP of protein-bound cofactors can also be estimated using sampling-based techniques, such as thermodynamic integration, which explicitly account for conformational dynamics.^32–35^ However, these methods are computationally expensive and therefore poorly suited for high-throughput applications. Determining cofactor RP through either experimental or conventional in silico strategies thus remains laborious, limiting their utility in large-scale protein design workflows. Moreover, due to the intrinsic complexity of Fe–S electronic structure, current computational tools still struggle to achieve high predictive accuracy.^34,36^

Recently, we demonstrated that ML provides a powerful alternative for predicting protein RP, leveraging its ability to analyze complex data sets and identify underlying patterns.^37^ Building on this, we developed FeS-RedPred, a ML platform for predicting the RP of Fe–S proteins, by adapting a previously developed model originally designed for flavoproteins. FeS-RedPred is a modular framework composed of multiple ML models, each built with a specific rationale, aimed both at optimizing predictive performance and at gaining insight into how structural features at different spatial scales influence RP. This effort is driven by the abundance of publicly available structural and electrochemical data on Fe–S proteins, which presents an opportunity for data-driven RP prediction. As an initial step, we focus on low-nuclearity Fe–S clusters, particularly rubredoxins and [2Fe–2S] proteins (including ferredoxin-, Rieske-, and mitoNEET-type clusters), as they offer an ideal training ground due to their well-characterized structures and redox properties. Beyond serving as controlled benchmarks for ML validation, these proteins are also biologically relevant, playing key roles in diverse metabolic and electron transfer pathways, oxygen resistance/protection mechanisms (as recently shown for nitrogenases),^200^ and other redox-driven cellular processes. In all these functions, activity relies on finely tuned RP, highlighting the need for accurate RP prediction in both basic and applied bioinorganic research.^38^ The promising performance of FeS-RedPred to metallocofactor redox prediction lays the groundwork for extending it to more complex Fe–S clusters and other inorganic cofactors.

## Results and Discussion

### Protein Data Set and Molecular Descriptors

To train, validate, and test FeS-RedPred, we compiled a curated data set of Fe–S proteins for which both structural data and experimentally determined RP are available, all identified through a systematic literature search updated as of July 2025 (Supporting Information File S2).^39–43^ The data set includes small Fe–S proteins containing either binuclear [2Fe–2S] clusters or mononuclear rubredoxin-type clusters. For each experimental structure included in the data set, the reported crystallographic resolution was retrieved from the Protein Data Bank (PDB) and is provided in an additional column of the data set. In cases where in silico mutants were generated (vide infra), the resolution of the corresponding reference PDB entry was assigned. The distribution of resolution values across the data set indicates that the large majority of structures are of high quality, with only a few entries above 2.5 Å (Figure S1a). Among the [2Fe–2S] proteins, coordination environments vary: ferredoxins display a canonical 4Cys coordination (two cysteines per iron), mitoNEETs adopt a 3Cys/1His arrangement, and Rieske proteins exhibit a 2His/2Cys coordination (Figure 2a). To increase the structural and functional diversity of the data set, we also included more complex metalloproteins and protein assemblies (e.g., [FeFe]-hydrogenases, xanthine oxidase, and dehydrogenases) that feature [2Fe–2S] clusters embedded in larger domains and/or cofactor networks. These entries expand the range of coordination environments and overall fold architectures, since we also include proteins showing atypical first-sphere ligation, such as 3Cys/1Asp coordination (as found in iron–sulfur flavoenzyme sulfide dehydrogenase) and 3Cys/1Arg coordination (as in biotin synthase).

**Figure 2:**
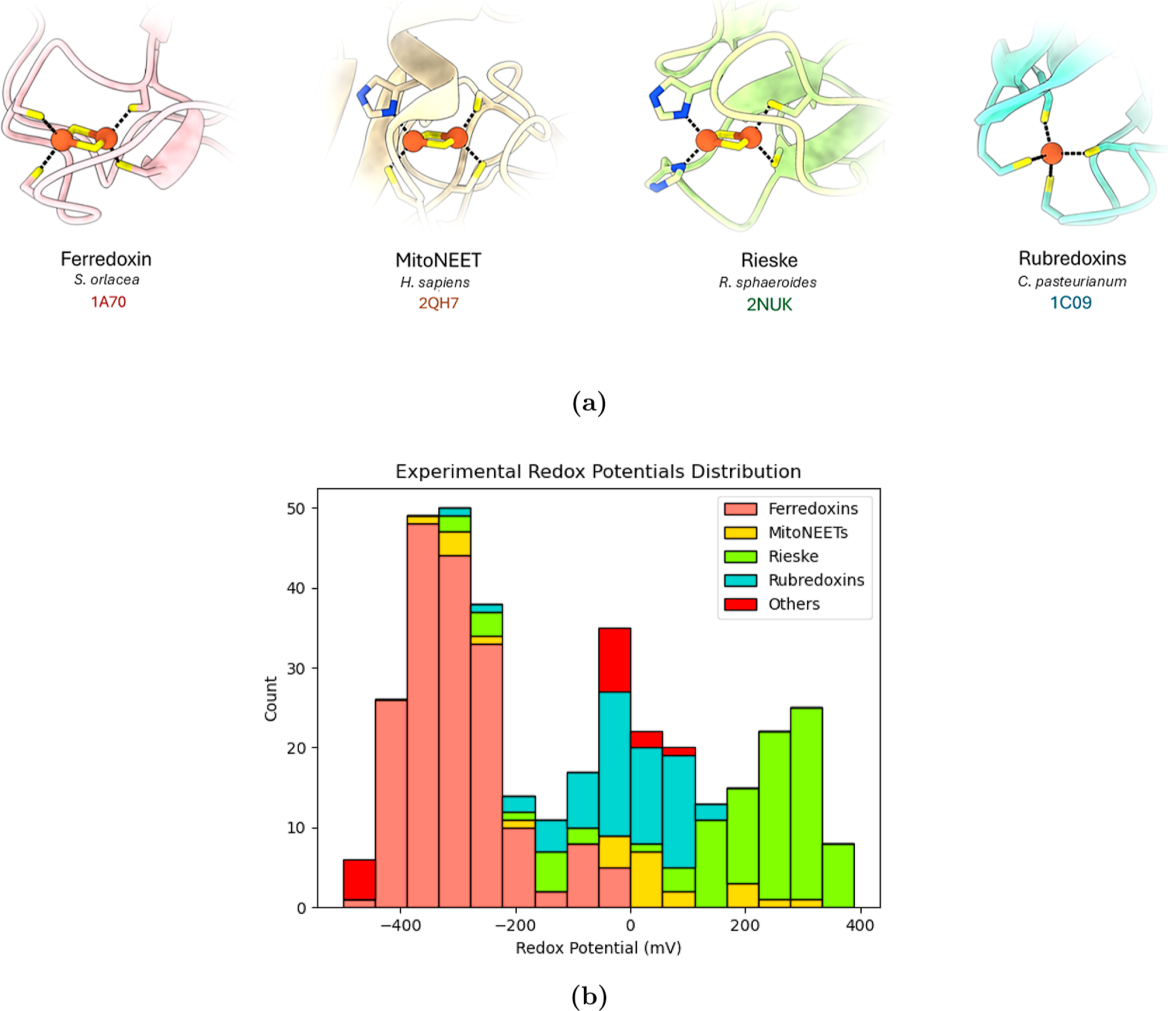
(a) Coordination of the Fe–S clusters in the four protein types considered. (b) Distribution of the RP for the proteins included in the data set, divided by protein type (“Others” indicate “non canonical” [2Fe–2S] cluster coordination, namely 3Cys/1Asp or 3Cys/1Arg).

In total, the data set comprises 59 proteins and their mutants, yielding 371 entries. Specifically, it includes 38 ferredoxin-type proteins and their mutants (177 entries); 3 mitoNEETs and their mutants (24 entries); 11 Rieske proteins and their mutants (93 entries); 4 rubredoxins and their mutants (61 entries); and 3 additional proteins with noncanonical [2Fe–2S] coordination, namely 3Cys/1Asp and 3Cys/1Arg configurations, and their mutants (16 entries in total). Mutations span the first, second, and outer coordination spheres, capturing a broad set of structural features known to influence RP modulation. When PDB structures for mutants were unavailable, we generated them in silico by introducing the mutation (starting from an available experimental structure of the same protein) and performing local minimization (see Methods). The selected proteins span a broad continuum of RP values, ranging from −460 mV to +390 mV (Figure 2b), ensuring sufficient coverage of the redox spectrum for robust ML training.

To effectively relate the 3D structure of a protein to its RP, it is essential to account for structural and environmental effects at different spatial scales. At short-range, the primary coordination sphere plays a dominant role, where factors such as ligand identity, hydrogen bonding with sulfide (S^2–^) or coordinating residues (cysteine or histidine), and other direct interactions significantly influence RP.^20,44,45^ At medium range, it is well established that residues in the second coordination sphere and beyond (outer sphere) can modulate the electronic properties of metal cofactors.^8,20^ Finally, long-range effects arise from the overall physicochemical properties of the entire protein, which contribute to redox tuning through electrostatic and structural organization. Notably, in small proteins (prevalent in our data set) medium- and long-range effects may overlap, as a significant portion of the protein falls within the distance thresholds used to define these influences.

To systematically capture these structural contributions, we designed a set of 66 molecular descriptors, inspired by the rationale outlined in Galuzzi et al.^37^ These features are automatically extracted from each protein’s PDB structure and are recalculated across three spacial ranges, defined as follows.

- Long-range descriptors (Figure 3, left) that encode global physicochemical properties of the entire protein, labeled as Protein.X, where X represents the specific feature analyzed.
- Medium-range descriptors (Figure 3, left), accounting for a protein portion within a sphere of radius *r*_1_ (set from 8 to 16 Å) centered at the Fe–S cluster’s barycenter, labeled as Bar.X;
- Short-range descriptors (Figure 3) that capture the local environment around each atom of the Fe–S cofactor, computed within a smaller radius *r*_2_ (3–5 Å, with *r*_2_ < *r*_1_), and labeled as CofAtom.Y.X, where Y specifies whether the atom is Fe or S (Figure 3, right).

**Figure 3:**
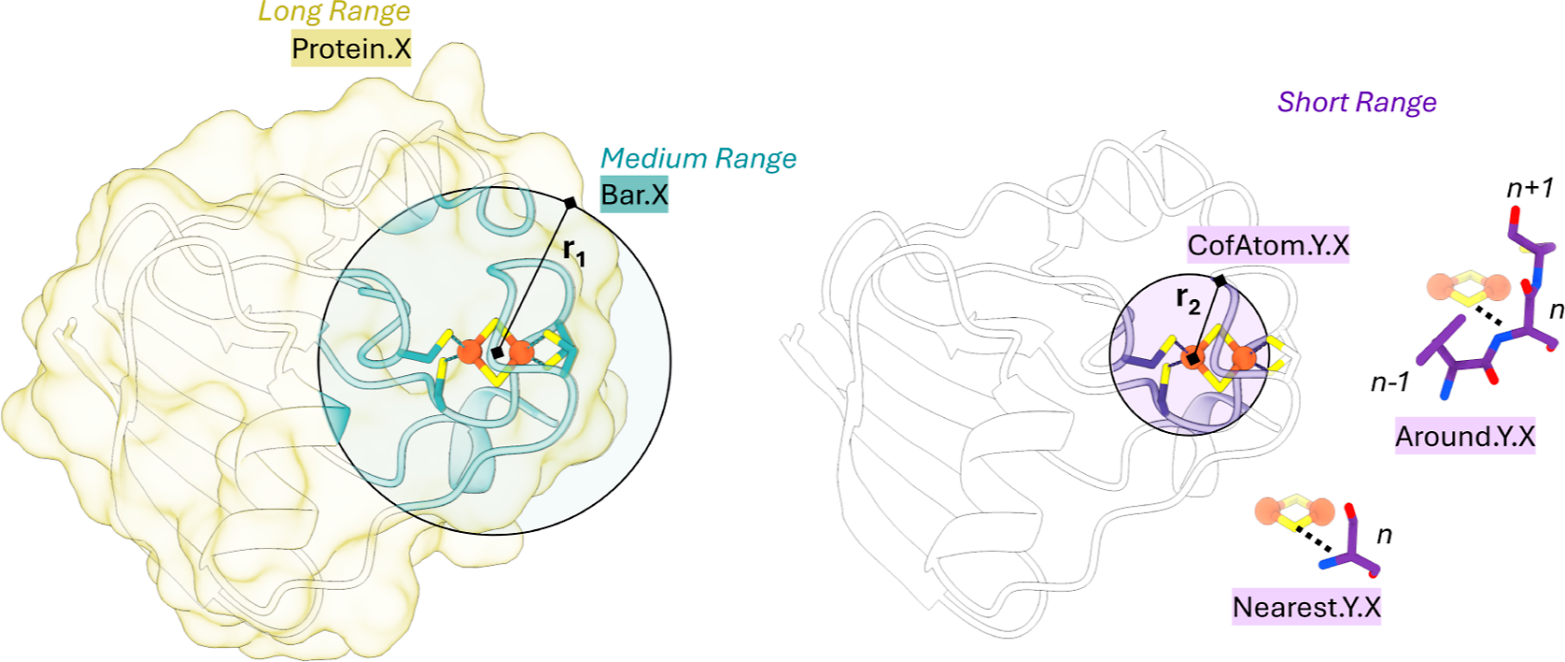
Schematic representation of 3D structure molecular descriptors used to develop the regression models, with long- and medium-range features shown on the left and short-range features on the right. For rubredoxins, where the cofactor consists of a single Fe ion, both *r*_1_ and *r*_2_ are centered on the Fe site.

Given that short-range effects are expected to have a stronger influence on RP, we further introduced two complementary sets of features describing the local environment around each cofactor atom. Specifically, we included descriptors representing the properties of the amino acid residue closest to each Fe or S atom of the cluster (NearestY.X, 28 descriptors) and descriptors that also account for the properties of the two sequentially adjacent residues of the nearest amino acid (AroundY.X, 32 descriptors).

Each descriptor category includes both count-based features (e.g., the number of residues with specific properties such as polarity or hydrophobicity) and summed parametrized property descriptors, taken from Abriata et al.^46^ The values of the descriptors depend on the chosen radii values, *r*_1_ and *r*_2_, so we calculated them for each combination of radii: *r*_1_ = 8, 9, ..., 16 Å and *r*_2_ = 3, 4, 5 Å. Since larger proteins may contain other inorganic/organic cofactors, we also introduced an ad hoc descriptor capturing cofactor type and multiplicity (i.e., the number of cofactors of a given type present within *r*_1_, *r*_2_ or in the whole structure).

Furthermore, since RP is sensitive to the pH at which it is measured,^47^ we included pH as a numerical feature in our model. When RP values were available for the same protein at multiple pH values, each condition was included as a separate entry in the data set. Altogether, this multiscale strategy yields a total of 765 features per protein structure. In addition, we accounted for variability arising from different experimental techniques, which can yield significantly different RP measurements even under the same conditions. For example, Zuris et al.^20^ reported a −25 ± 4 mV systematic shift between values obtained via protein film voltammetry and those from optical methods, comparable to the Mean Absolute Error (MAE) of our models. To address this variability, we developed a variant of our ML model that includes the measurement technique as a categorical feature, allowing the model to adjust predictions based on methodological context (see below).

### FeS-RedPred Performance

In our previous work, we explored the possibility of using efficient ML models for the prediction of protein RP.^37^ More in detail, we performed a broad comparison of different models (Extreme Gradient Boosting (XGB), Random Forest, Decision Tree, SVM and Logistic Regression) and carried out an extensive hyperparameter optimization through a grid search strategy for any model. Based on these previous results, we selected XGBoost as the best-performing approach for the present study and Linear Regression (LR) as a baseline control.

To optimize both computational cost and model performance, we trained different models based on the following strategies.

**A-**adopting the same approach used in Galuzzi et al.,^37^ i.e., using all the descriptors described above, calculated for each combination of *r*_1_ and *r*_2_. These models are referred to as **A-**XGB and **A-**LR (Figure 4, left).
**B-**simultaneously considering all features across all combinations of radii, to avoid redundancy. These models are referred to as **B-**XGB and **B-**LR (Figure 4, right).

**Figure 4:**
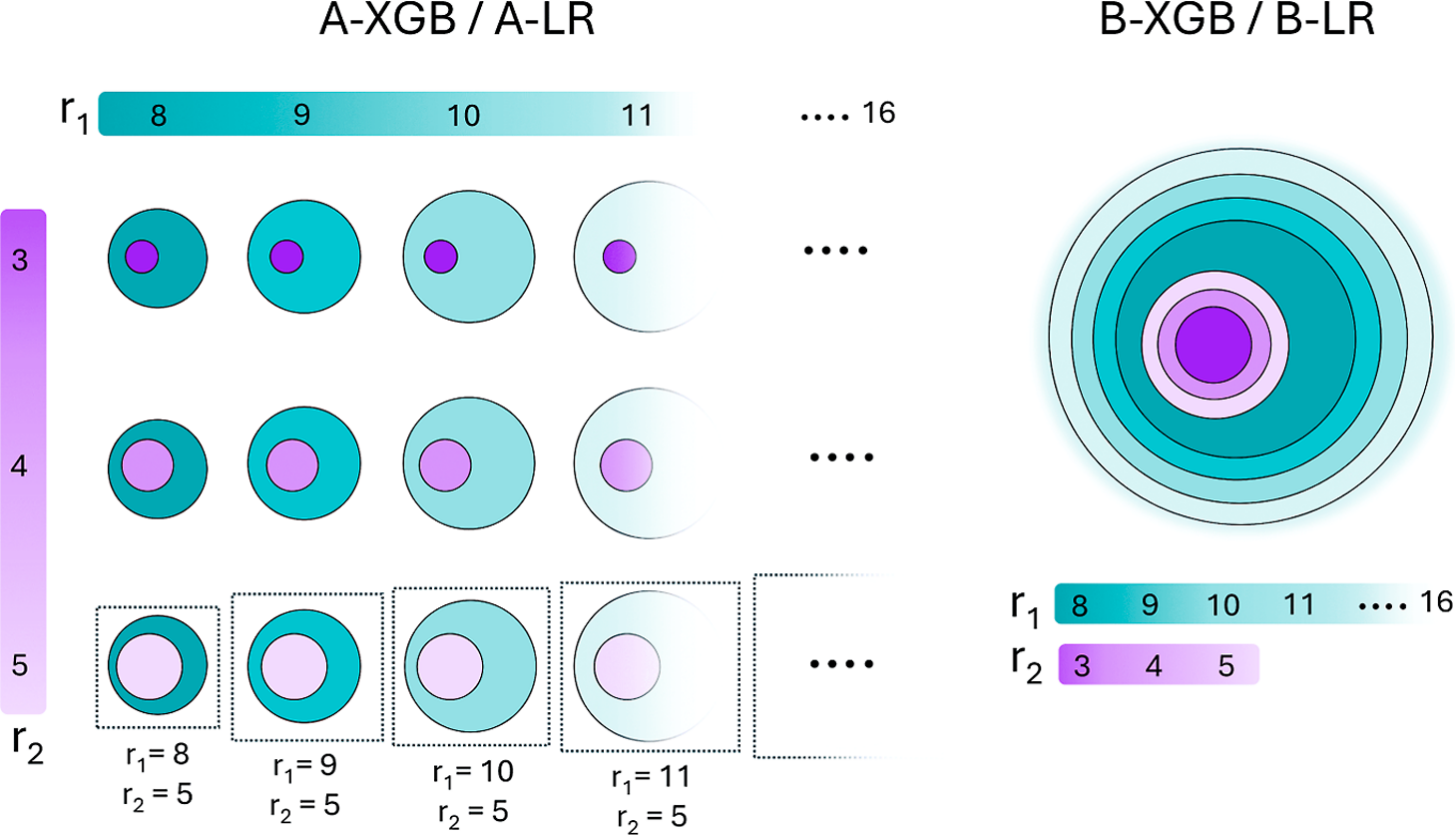
Strategies used to train FeS-RedPred. In the A-XGB/A-LR approach (left), a separate model is trained for each specific combination of radii (*r*_1_, *r*_2_). Features are recalculated for each pair, meaning that descriptors at a given *r*_1_ are computed multiple times to be combined with different values of *r*_2_. In the figure, individual models with fixed $r_{2}=5\;Å$ and varying *r*_1_ are highlighted with separate boxes. In the B-XGB/B-LR strategy (right), a single model is trained by simultaneously incorporating features computed across all *r*_1_ and *r*_2_ combinations, thus reducing redundancy.

Table 1 summarizes the performance of these approaches, reporting results for the optimal radii combination when applicable, evaluated on an unseen subset of data (see Methods for details). The XGB hyperparameters are tuned using a grid search strategy, where the grid was specifically designed to reduce the overfitting effect in our data set where the number of descriptors exceeds the number of examples. Moreover, hyperparameters were optimized using the training set only without using any data from the test set during the performance assessment (see Methods for details).

**Table 1:** Performance Metrics (MAE, Root Mean Squared Error (RMSE), R2, SC, and Execution Time) for Different ML Models

model	MAE (mV)	RMSE (mV)	*R* ^2^	SC	time of execution (h:min:s)
**A-**XGB *r*_1_ = 11 *r*_2_ = 4	39.9 ± 4	57.7 ± 8	0.94 ± 0.02	0.97 ± 0.01	22:38:53
**B-**XGB	40.5 ± 5	60.1 ± 11	0.94 ± 0.03	0.95 ± 0.02	2:44:57

Model **A-**XGB achieved excellent performance, with a MAE of approximately 39.9 mV for the best-performing radius combination and an *R* ^2^ of 0.94 (Table 1). Similar results were observed for other radius combinations, as shown in the heatmap in Figure 5, with limited variability in performance, which remained just above 40 mV, well below 1 kcal/mol. The performance of the **A-**LR model confirms that a linear relationship is less suited to capture the complex protein–cofactor interplay governing RP.

**Figure 5:**
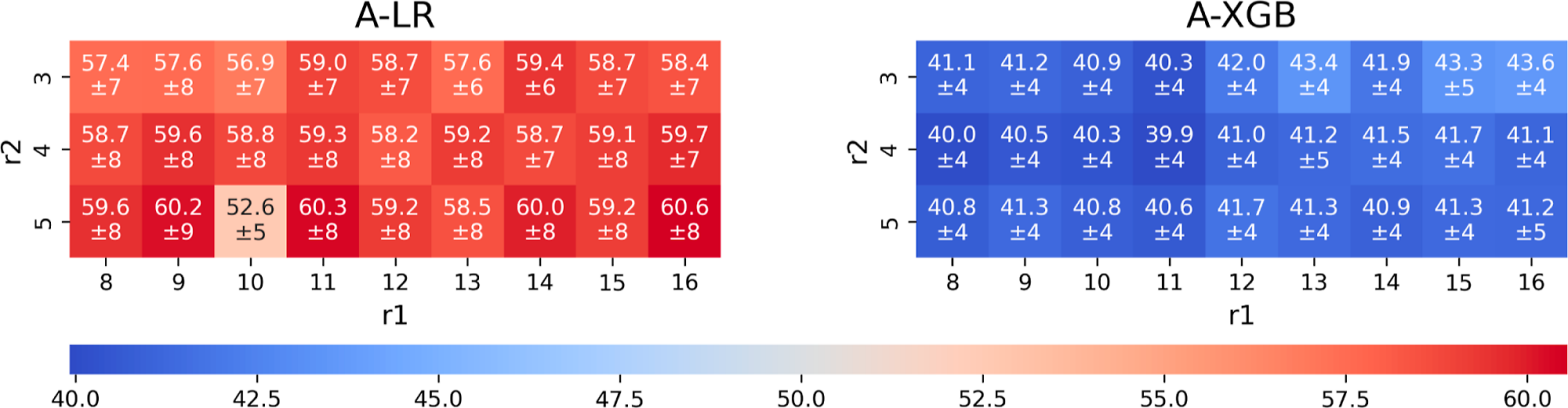
Mean and standard deviation of MAE as a function of *r*_1_ and *r*_2_. All the values are expressed in mV.

Although the different experimental methodologies can introduce variability in RP measurements, including this information as a descriptor did not significantly improve model accuracy while increasing computational cost, and appears to offer limited predictive value for novel proteins (see Supporting, Table S1 for details). For this reason, we did not include this approach in further analyses. Interestingly, considering simultaneously all the radii combinations in **B-**XGB, not only maintained excellent predictive performance but also significantly reduced computational costs (Table 1).

Figure 6 reports the MAE for the different protein types considered for **A-**XGB and **B-**XGB. As expected, MitoNEETs, being the least represented proteins in the data set, exhibit the highest prediction error. Interestingly, despite the fact that the data set contains more Rieske proteins than rubredoxins, the latter are predicted with similar accuracy. This may be attributed to the decision-tree-based nature of XGB, which benefits from the unique structural features of rubredoxins compared to the other protein classes. Nevertheless, the experimental vs predicted RP correlation remains high across all protein types, and the observed errors remain within acceptable limits (Figure 6).

**Figure 6:**
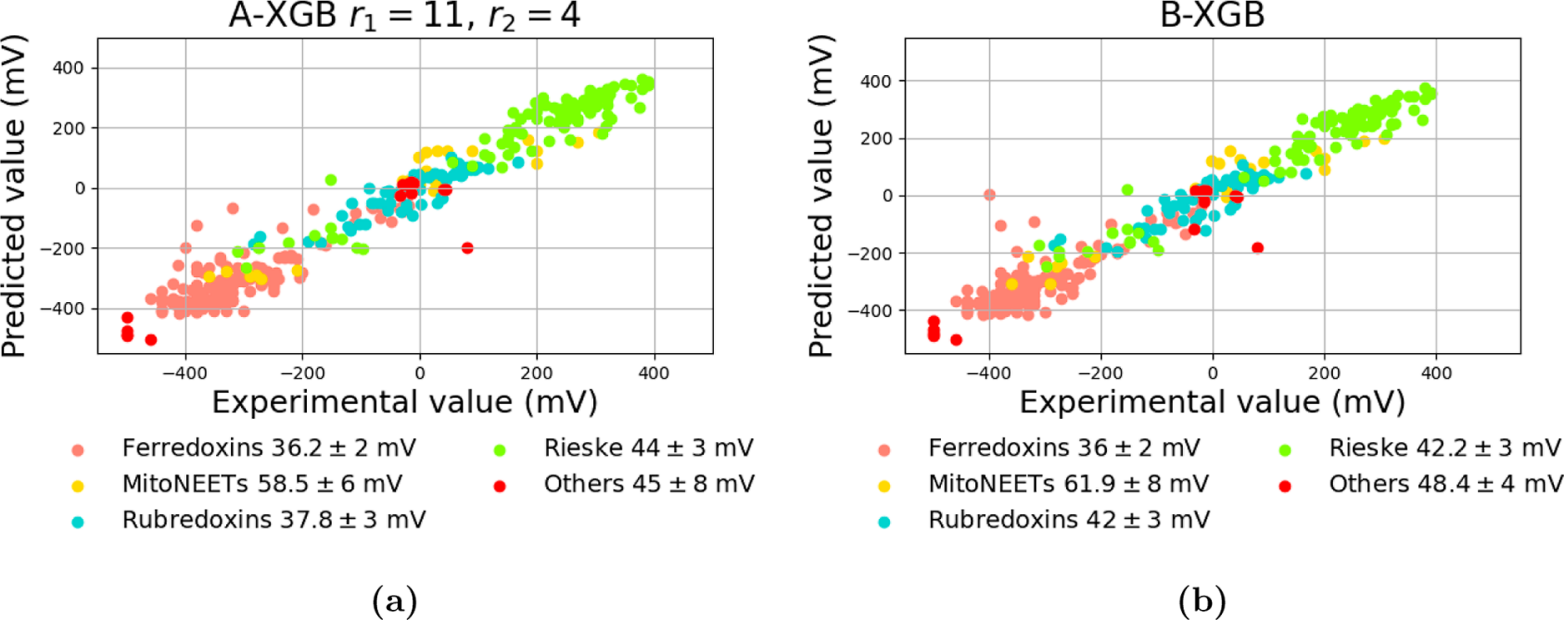
Predicted VS experimental RP for A-XGB model (a) and B-XGB (b). MAE values (in mV) are reported for each protein type.

To further evaluate the predictive capabilities of our model, we conducted two complementary tests. In the first test, one protein out of every 37 in the data set (10 proteins in total) was entirely excluded from training. The model trained on the remaining entries was then used to predict the RP of the excluded proteins de novo. The model achieved consistently low errors, with the vast majority of proteins deviating by less than 30 mV from experimental values, and an overall mean absolute error of 28 mV (Figure S2). These results indicate that the model can reliably predict RP for proteins not included in our data set.

In the second test, we applied the same strategy but excluded a set of mutants for individual proteins, selecting two representatives per protein class (depending on the available data) to increase, decrease, or leave the RP essentially unchanged relative to the wild type. Here, the model not only achieved small prediction errors, averaging 12.5 mV across the selected mutants, but also successfully reproduced the expected directional changes in RP (Figure S3).

Taken together, these results further support the potential use of our tool in protein design, as it can predict not only the magnitude but also the direction of RP shifts in designed mutants. Remarkably, in all these cases, the prediction of the RP for a single protein required less than 1 s.

A closer inspection of the prediction errors revealed two proteins that consistently emerge as major outliers across all models (Figure 6). The first case corresponds to the [2Fe–2S] ferredoxin from *Escherichia coli* quinol–fumarate reductase (PDB ID: 1L0V, C62S mutant), in which replacement of a cysteine ligand by serine causes an exceptionally large redox shift (−322 mV vs −79 mV in the wild type).^48^ This effect is far more pronounced than in other single-Cys-to-Ser mutants of the same protein (−182, −110, and −49 mV), and prediction absolute errors scale accordingly (≈250, 90, 60, and 30 mV, respectively). We attribute this limitation to the very low representation of such coordination motifs in the training set (only 10 entries, including four rubredoxin mutants), which likely prevents the model from adequately capturing such extreme and heterogeneous shifts. The second outlier is the [2Fe–2S] cluster located in the NADH-dependent ferredoxin:NADP oxidoreductase (NfnI) from *Pyrococcus furiosus* (PDB ID: 5JCA).^49^ This cluster is coordinated by an aspartate ligand, a motif that is also poorly represented in the data set (11 entries), and displays an unusually high RP (+80 mV). Moreover, it has been suggested that NfnI undergoes conformational rearrangements at the NfnA/NfnB interface, which alter the cluster environment and facilitate electron bifurcation.^50,51^ Such dynamic effects, involving multiple conformational states in solution, are not captured by our current static-descriptor approach and may therefore contribute to the observed discrepancy. Together, these cases highlight two intrinsic limitations of the present framework: (i) insufficient representation of uncommon coordination environments in the training set, and (ii) the lack of an explicit treatment of protein dynamics and conformational ensembles. Both aspects represent promising directions for future model refinement.

Interestingly, we did not observe any correlation between prediction errors and crystallographic resolution, indicating that poorly determined structures are not significantly biasing the data set (Figure S1b). This robustness is consistent with the way our descriptors are defined, i.e. simple, local, and based on physicochemical features within fixed cutoff radii rather than precise atomic coordinates, and aligns with similar observations reported by Min et al.^52^

### Molecular Descriptor Analysis

To gain a deeper understanding of how protein structure influences RP, we assessed the contribution of individual molecular descriptors to the model’s predictions. Identifying the most relevant features is not only essential for interpreting the model but also provides valuable insights that could guide protein design and engineering. To achieve this, we performed a SHAP (SHapley Additive exPlanations) analysis, focusing on the **A-**XGB model, also to examine how feature importance changes with *r*_1_ and *r*_2_, i.e. the radii used to define structural descriptors. Figure 7 summarizes the top-ranking molecular descriptors driving RP prediction across different cutoff radii (Figure 7a), along with their Spearman correlations with RP (Figure 7b). The same top-ranked descriptors are also obtained for the **B-**XGB model (see Supporting Information, Figure S5 for details).

**Figure 7:**
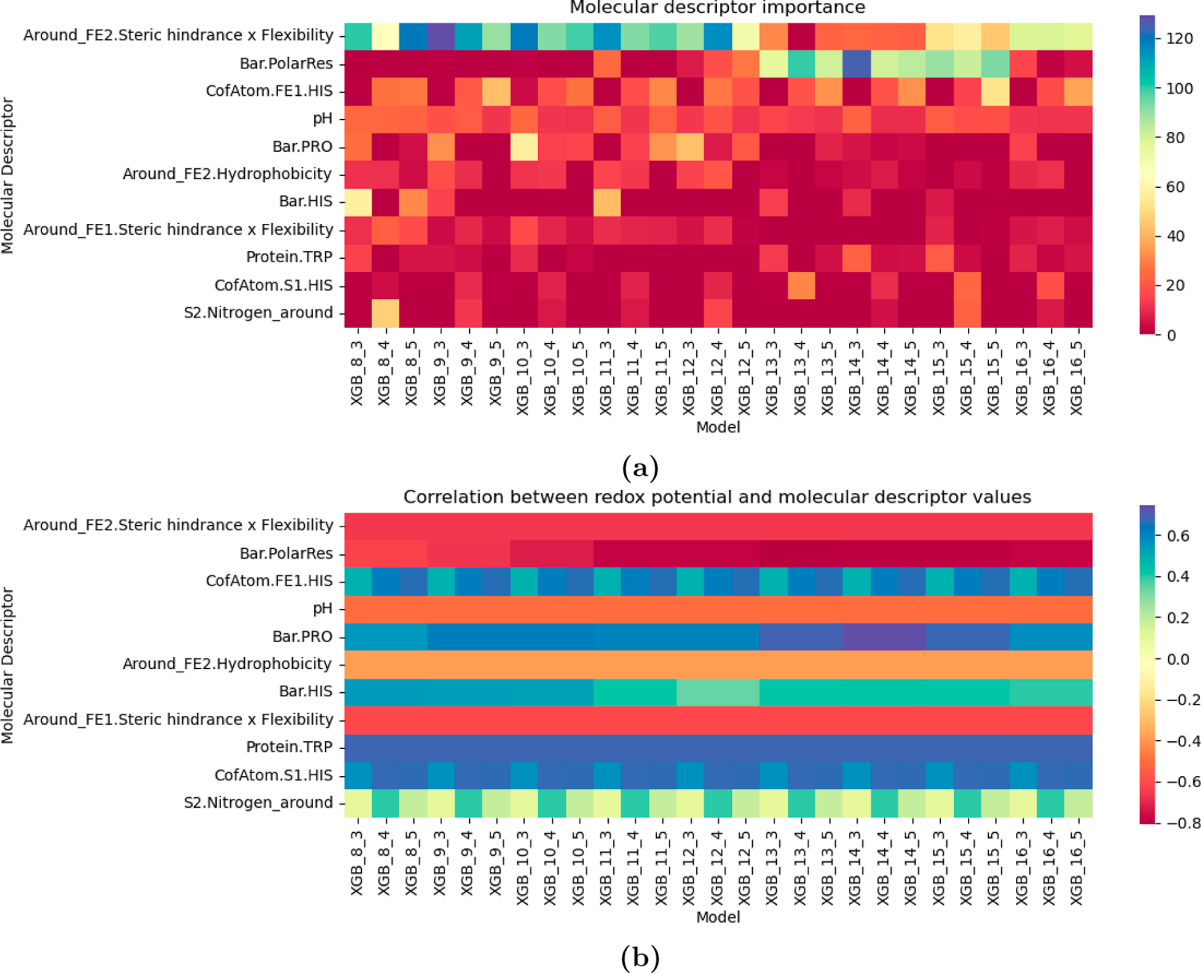
**(a)**. Heatmap of the molecular descriptor importance. Higher values indicate a greater relevance of the descriptor in the model. The molecular descriptors are ordered based on the average importance value across all models. **(b)**. SC between RP and molecular descriptor values. Values close to −1 indicate a negative correlation (higher molecular descriptor values correspond to lower RP and vice versa), while values near +1 indicate a positive correlation.

Where feasible, we attempted to rationalize their impact based on known physicochemical principles. While some descriptors are nontrivial to interpret due to their composite nature, others are difficult to link directly to RP from a chemical perspective and may instead correlate indirectly with structural or environmental effects. Nonetheless, we selected a few top-ranked descriptors for closer inspection to explore whether chemically reasonable insights could be extracted and to better understand how the model captures structure–function relationships. Among the most impactful features is one that combines steric hindrance and backbone flexibility of the three residues closest in sequence to Fe. This descriptor exhibits a strong negative correlation with RP, meaning that its increase tends to shift RP to more negative values. Although its formulation does not directly map to a single physical property, residue-level analysis reveals that cysteine and serine contribute most to higher descriptor values, aligning with their known influence in lowering RP when coordinating Fe.^20,45^

The second-ranked descriptor is the count of polar residues around the cluster. It is well established that the polarity of the surrounding environment can influence RP. For example, the unusually positive RP of the rubredoxin-type cluster in rubrerythrin has been attributed to the substitution of Val residues (common in rubredoxins) with polar side chains, which are thought to substantially modulate the local electrostatic environment at the redox site.^8^ However, a closer examination of our data set suggests that this descriptor, in this specific case, likely reflects first-sphere effects rather than independent second-sphere contributions. In our scheme, “polar residues” include only neutral polar side chains, and this feature is not consistently ranked as important across all cutoff radii. Its apparent relevance arises only in certain models (Figure 7a), where it occasionally swaps rank with the top-ranked feature, which is associated with the number of cysteines coordinating the Fe center. Since cysteines are classified as neutral polar residues while histidines, lysines, and arginines are grouped as basic/positively charged residues, these two features capture overlapping information. In practice, both primarily report the presence of coordinating cysteines, meaning that interpreting “polar residues” as an independent second-sphere determinant would be misleading.

Several of the remaining top-ranked descriptors are related to the presence of histidine residues or nitrogen-containing atoms near Fe or S atoms. While some directly quantify the number of nearby His residues, capturing known redox shifts caused by His replacing Cys in first-shell coordination, as seen going from ferredoxin, to NEET, and finally to Rieske proteins, others more generally account for local nitrogen density. These features may also reflect a combination of effects beyond His ligation, such as electrostatic stabilization of the reduced state by protonatable residues (such as Lys and Arg), and hydrogen bonding with S^2–^ ligands. This latter contribution is consistent with previous computational and experimental studies on both binuclear Fe–S clusters and rubredoxins, which have highlighted the role of hydrogen bonding in modulating RP of both rubredoxins and [2Fe–2S] proteins.^44,53,54^

Additionally, pH emerges as a significant factor, with lower pH values being associated with more positive RP, as expected. Taken together, these findings confirm that the model effectively captures the complex interplay of local steric effects, electrostatics, ligand identity, and environmental factors. However, these findings also underscore the difficulty of disentangling first- and second/outer-sphere contributions in our data set. First-sphere effects dominate the redox behavior of Fe–S clusters, making it challenging to isolate the influence of more distal determinants. In contrast, in our previous study on flavoproteins,^37^ the absence of metal coordination allowed for a more straightforward interpretation of feature importance. Resolving subtler second/outer-sphere effects in Fe–S proteins would ideally require training models on proteins with identical first-sphere coordination, so that differences could be directly attributed to the surrounding environment. Currently, the limited number of available entries per cluster type prevents this without compromising predictive performance, but efforts in this direction are already underway in our group.

### Descriptor Contributions Across Multiple Spatial Scales

To systematically assess the impact of molecular descriptors at different spatial scales, we trained multiple XGB models by selectively excluding descriptors associated with short-, medium-, and long-range effects.

- No short-range: excludes descriptors around individual cofactor atoms.
- No medium-range: excludes descriptors around the cofactor barycenter.
- No long-range: retains only short- and medium-range descriptors, excluding whole-protein features.

To further disentangle the contribution of each descriptor type, we trained separate models using only one category of molecular descriptors at a time.

- Short-range only: uses only descriptors around the cofactor atoms.
- Medium-range only: uses only descriptors around the cofactor barycenter.
- Long-range only: uses only whole-protein descriptors.

As expected, removing any set of descriptors results in a decline in predictive accuracy (Table 2 and Figure 8). The difference in performance of these models were confirmed by a pairwise statistical analysis based on the Mann–Whitney U rank test. All the obtained *p*-values of the statistical analysis are reported in the Supporting Information (Figure S6). This trend highlights the importance of integrating features across multiple spatial scales to achieve accurate RP predictions. The long-range only model performs the worst, confirming that whole-protein features alone are insufficient to provide efficiency. Conversely, models retaining short-range descriptors perform significantly better, underscoring the importance of local interactions. However, the overall performance drop observed along the series is not dramatic (∼8 mV). This can be reasonably explained by the intrinsic overlap between local and global descriptors: residues near the cofactor contribute to both short-range and protein-wide features, meaning that some local information is inherently embedded in global descriptors. Nonetheless, explicitly including short-range features proves more effective, both in terms of accuracy and in capturing the chemically and physically relevant environment of the cofactor. The SHAP analysis confirms that the most influential features in these models align with those identified in the full **A-**XGB model. Full SHAP analysis results for these models are provided in the Supporting Information, Figures S7–S12.

**Table 2:** Performance Metrics (MAE, *R* ^2^) for Different ML Models

XGB model	long-	medium-	short-	MAE (mV)	*R* ^2^
**A-**XGB *r*_1_ = 11, *r*_2_ = 4	X	X	X	39.9 ± 4	0.94 ± 0.02
no long-range *r*_1_ = 11, *r*_2_ = 4		X	X	40.2 ± 5	0.94 ± 0.02
no medium-range *r*_2_ = 4	X		X	41.7 ± 4	0.94 ± 0.02
no short-range *r*_1_ = 8	X	X		42.6 ± 6	0.92 ± 0.04
short-range only *r*_2_ = 5			X	42.6 ± 5	0.93 ± 0.02
medium-range only *r*_1_ = 15		X		44.4 ± 7	0.91 ± 0.04
long-range only	X			47.6 ± 5	0.91 ± 0.03

**Figure 8:**
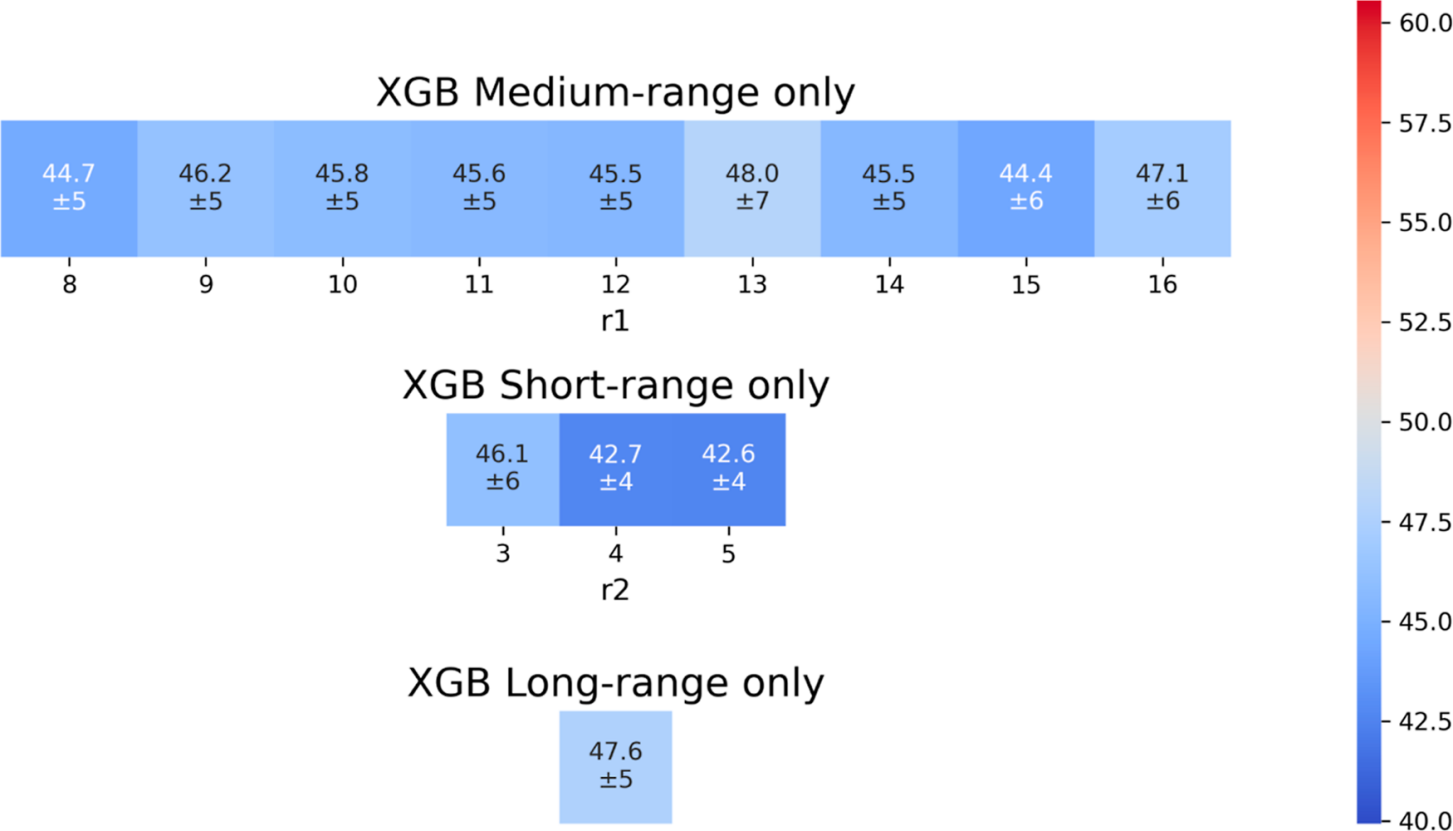
Mean and standard deviation of MAE (in mV) as a function of *r*_1_ and *r*_2_.

## Conclusions

In this work, we presented FeS-RedPred, a ML framework adapted from a model we originally developed for flavoproteins. By designing a set of structure-based descriptors that encode the 3D environment surrounding the cofactor, and applying the same underlying rationale, we demonstrate that the method can be extended across different classes of redox-active proteins. We further showed that our approach can be optimized in terms of efficiency by reducing descriptor calculation redundancy, which lowers computational cost without sacrificing predictive accuracy. In addition, it can be seamlessly extended to include features such as the presence of other cofactors or categorical variables, as, e.g., the experimental technique used to determine RP. However, in our data set, none of these additional features has a dominant influence on the predicted response.

A recent high-level benchmark by Jafari et al. systematically evaluated different computational strategies for predicting RP in 12 representative Fe–S proteins. Their best-performing protocol (QM/MM geometries followed by QM + COSMO single-point corrections, ε = 80) achieved very high accuracy for certain cluster classes, such as Rieske centers, while showing larger deviations for others, including rubredoxins and [2Fe–2S] ferredoxins. After correcting for a systematic shift of −0.55 V, the authors reported mean absolute and maximum deviations of 0.17 and 0.44 V, respectively. In terms of resources, this protocol requires moderate computational cost (∼95 CPU hours on 20 cores). In comparison, FeS-RedPred reaches an average error of ∼40 mV across different cluster classes, without requiring scaling factors and relying only on simple descriptors directly derived from PDB structures. For proteins that overlap with the Jafari et al. benchmark, our method attains an average error of 15.4 mV, with particularly low values for all systems (5–29 mV). Importantly, model training is computationally lightweight (ranging from less than 3 h to at most 1 day depending on the configuration, Table 1), and once trained, predictions on new proteins can be obtained within seconds. This makes the approach particularly suitable for rapid and large-scale applications and competitive with higher-level methods in terms of efficiency.^36^ FeS-PredRed resulting performance is stable, with low variance across folds, and strongly suggesting that the model captures genuine structure–function relationships rather than noise. Moreover, it bypasses the complications of explicitly modeling the antiferromagnetic coupling that characterizes the electronic structure of Fe–S clusters in standard DFT calculations.

Interestingly, Min et al.^52^ recently showed that a small set of structural descriptors, such as total charge and average Fe valence, can capture global trends across different cluster types using linear regression on a relatively limited data set of Fe–S proteins. While the two approaches differ substantially in methodology and scope, it is nevertheless informative to provide some contextual comparison. For the simpler mono- and binuclear clusters, their model achieves an average discrepancy of ∼80 mV, only slightly higher than the 57–60 mV obtained when applying linear regression to our own data set (Figure 5), but above the ∼40 mV reached with the XGB implementation of FeS-RedPred (Figure 5, Table 1). This comparison highlights that extending the set of protein-encoded features and adopting nonlinear models are key elements for achieving higher predictive accuracy. At present, FeS-RedPred has been applied to mononuclear and binuclear [2Fe–2S] clusters. This deliberate focus was motivated by the novelty of our approach, allowing us to train and benchmark the ML framework in a controlled setting and to gain confidence in its performance before moving to more complex cases. Notably, the same framework has also proven effective on flavoproteins with comparable accuracy,^37^ supporting its potential broader generalizability across different cofactor chemistries. Extending the approach to higher-nuclearity clusters, such as [3Fe–4S] and [4Fe–4S], therefore represents a natural next step, and will likely further broaden its applicability to Fe–S proteins of technological and bioenergetic relevance^55–59^ In this regard, it is worth noting that the average error reported by Min et al.^52^ increases to ∼120 mV when evaluating performance on their full data set, which also includes [3Fe–4S] and [4Fe–4S] proteins. Therefore, we cannot exclude that a similar loss of performance might also occur in our case upon extending the model to higher-nuclearity systems. On the other hand, such an extension would naturally enlarge and diversify the training set, which could in turn counterbalance potential accuracy losses, since data set size and diversity are key factors in the performance of ML models. Work along this line is already underway in our group, and current results provide a solid groundwork for such future developments.

Beyond its predictive performance, our model also provides interpretable outputs, although in Fe–S clusters the dominant first-sphere effects make second-sphere contributions difficult to disentangle. Future work on larger data sets with controlled first-sphere coordination will be key to better resolving these subtler influences.

Overall, FeS-RedPred provides a versatile and efficient tool for supporting both the design of artificial Fe–S proteins and the engineering of native ones.^60–64^ This enables a data-driven approach to protein engineering, helping to prioritize mutations for experimental validation without relying solely on trial-and-error strategies. Specifically, the model may be applied to (i) rapid screening of large mutant libraries to identify the most promising variants; (ii) guiding protein design by highlighting mutations likely to shift RP; and (iii) assisting in the assignment of ambiguous RP, e.g. in proteins containing multiple Fe–S clusters.

Future developments will also include the consideration of computationally predicted structures, both to enrich the data set and to test model performance on proteins lacking experimentally determined structures. In particular, the revolutionary advances in structure prediction achieved in recent years (e.g., AlphaFold, RoseTTAFold) now provide models with confidence levels that in some cases approach those of experimental structures. Moreover, we aim to explicitly incorporate protein dynamics and conformational ensembles, which in certain cases can significantly influence RP. Finally, with the growing availability of structural and electrochemical data across metalloprotein families, such as copper proteins and cytochromes, this framework holds promise as a broadly applicable platform for advancing the design of novel electron transport networks and biocatalyst optimization.

## Methods

### In Silico Mutant Generation

All the 3D structures for the wild-type proteins of our data set were downloaded from the PDB. When a PDB structure for a given mutant was unavailable, we generated it in silico with the BioLuminate^65^ suite within Maestro.^66^ First, we prepared the wild type protein using the PrepWizard tool in Maestro. To generate the mutants, we employed the residue and loop mutation tool in Maestro, followed by localized minimization within a 5 Å radius of the mutation site. All mutants generated with this procedure differ from the wild-type protein by one or two amino acid substitutions, starting from an available experimental structure of the same protein. To validate the adequacy of this approach, we selected ferredoxin 1FXA, the protein in our data set with the largest number of mutants (20), and evaluated stability changes upon mutation. ΔΔ*G* values calculated with FoldX^67^ were mostly neutral (within ±1 kcal/mol), with only a few slightly above this threshold. Consistent results were obtained with two additional predictors, DynaMut and DDGun (Figure S13).^68,69^ Together, these analyses indicate that the mutations introduce only local perturbations, as expected, and that our local minimization strategy provides an adequate structural treatment for the generated mutants. To further justify the inclusion of these in silico–generated mutants in our data set, we also trained a model using only experimental structures. This led to a substantial reduction in data set size (137 entries) and, as a consequence, to a decrease in predictive performance, with the MAE increasing to 53 mV (Table S1). This outcome confirms that including a larger number of entries and structural variability is essential to maintain high predictive accuracy, as expected.

### The ML Model

We used a decision-tree-based ensemble method called Gradient Boosting (GB) as an ML regressor.^70^ GB is an ensemble learning technique commonly used for regression or classification tasks. It works by sequentially building a model that corrects the errors and improves the previous models. The approach begins with a simple base model, often a shallow decision tree, which predicts the target variable. The difference between the actual and predicted values, known as residuals, is calculated, and a new model is trained to predict these residuals. This process repeats iteratively, where each new model adds to the prediction of the previous ones, reducing the overall error in a step-by-step manner. The model’s predictions are combined to form the final output, and the algorithm seeks to minimize the loss function through gradient descent, ensuring that the errors are progressively reduced.

XGBoost (Extreme Gradient Boosting)^71^ is an optimized implementation of GB, designed to be faster and more efficient, especially for large data sets and complex regression tasks. XGBoost incorporates several key improvements over traditional gradient boosting, such as regularization to reduce overfitting, parallelization for faster training, and a more efficient tree-pruning strategy. These features make XGBoost highly accurate and scalable, making it a popular choice for regression problems.

Notably, since the number of descriptors calculated for each protein structure exceeds the number of training samples, we adapted the ML model, which usually has the default values of hyper-parameters suitable for a data set with a high number of examples. More in detail, we carefully designed the hyperparameter grid to explore values that promote model regularization. In particular, we tuned the number of estimators (n_estimators), and the tree depth (max_depth), which collectively control the model’s complexity and generalization capacity.

### The ML Experimental Setup

For all trained and tested models, we used the same ML pipeline previously adopted in Galuzzi et al.^37^ In particular, we implemented a nested cross-validation strategy, consisting of a 5-fold outer cross-validation for model evaluation and a 10-fold inner cross-validation for hyperparameter tuning of the XGBoost algorithm.

We used a k-fold cross-validation approach instead of a simple train-test split to reduce the risk that the results depend too heavily on a particular subset of the data.

In the inner loop (i.e., for hyperparameter tuning), the model was trained on 9 folds and validated on the remaining one, cycling through all folds. Hyperparameters were optimized using a grid search strategy aimed at minimizing the Mean Absolute Error (MAE). In particular, we tested three different values (3,4, and 5) for the maximum depth of the trees, three different values (100, 150, and 200) for the number of gradient boosted trees, four different values (0.01,0.1,0.2, 0.4) for the learning rate, and three different values (1,5, 10) for minimum sum of instance weight needed in a child node for a split to be made. Therefore, we tested a total of 108 different possible hyperparameter configurations.

In the outer loop (i.e., for model evaluation), the tuned model was trained on 4 folds (≈80% of the data set) using the best hyperparameters identified in the inner loop, and then tested on the remaining fold (≈20%). This procedure was repeated until each fold had served once as the independent test set. Importantly, this guarantees that every protein in the data set is evaluated de novo, i.e. without the model ever having seen it during training, thus providing an unbiased assessment of generalization performance. To ensure robustness against performance variability due to random data splitting, the entire nested cross-validation procedure was repeated 10 times, allowing for a comprehensive assessment of model stability and generalization performance.

## Supplementary Material





## Data Availability

The code and data used in this work are publicly available at the GitHub repository: https://github.com/CompBtBs/FeS_RedPred.

## Notes

The Supporting Information includes: **File S1** -Additional outputs and analysis details. This file contains detailed performance metrics (MAE, RMSE, *R* ^2^, SC, and execution time) for the model using the experimental methodology for measuring RP as a categorical input feature. Additionally, it provides SHAP analyses highlighting the most important molecular descriptors for each trained model and their correlation with the predicted RP (PDF)
**File S2**-Fe–S protein data set and descriptors details. This file contains the complete data set, the feature sets used for the best model of each training type, and the full performance metrics for each training configuration (XLSX)

## References

[1] Stripp S. T., Duffus B. R., Fourmond V., Léger C., Leimkühler S., Hirota S., Hu Y., Jasniewski A., Ogata H., Ribbe M. W. (2022). Second and Outer Coordination Sphere
Effects in Nitrogenase, Hydrogenase, Formate Dehydrogenase, and CO
Dehydrogenase. Chem. Rev..

[2] Addison H., Pfister P., Lago-Maciel A., Erb T. J., Pierik A. J., Rebelein J. G. (2025). Two Key Ferredoxins
for Nitrogen Fixation Have Different
Specificities and Biophysical Properties. Chem.
Eur. J.

[3] Rodríguez-Maciá P., Kertess L., Burnik J., Birrell J. A., Hofmann E., Lubitz W., Happe T., Rüdiger O. (2019). His-Ligation
to the [4Fe–4S] Subcluster Tunes the Catalytic Bias of [FeFe]
Hydrogenase. J. Am. Chem. Soc..

[4] Fasano A., Fourmond V., Léger C. (2024). Outer-sphere
effects on the O2 sensitivity,
catalytic bias and catalytic reversibility of hydrogenases. Chem. Sci..

[5] Tse E. C. M., Zwang T. J., Barton J. K. (2017). The Oxidation
State of [4Fe4S] Clusters
Modulates the DNA-Binding Affinity of DNA Repair Proteins. J. Am. Chem. Soc..

[6] Honarmand
Ebrahimi K., Ciofi-Baffoni S., Hagedoorn P.-L., Nicolet Y., Le Brun N. E., Hagen W. R., Armstrong F. A. (2022). Iron–sulfur
clusters as inhibitors and catalysts of viral replication. Nat. Chem..

[7] Heffner A. L., Maio N. (2024). Tip of the Iceberg:
A New Wave of Iron–Sulfur Cluster Proteins
Found in Viruses. Inorganics.

[8] Luo Y., Ergenekan C. E., Fischer J. T., Tan M.-L., Ichiye T. (2010). The molecular
determinants of the increased reduction potential of the rubredoxin
domain of rubrerythrin relative to rubredoxin. Biophys. J..

[9] Burgess B. K., Lowe D. J. (1996). Mechanism of Molybdenum
Nitrogenase. Chem. Rev..

[10] Hurley J. K., Weber-Main A. M., Stankovich M. T., Benning M. M., Thoden J. B., Vanhooke J. L., Holden H. M., Chae Y. K., Xia B., Cheng H., Markley J. L., Martinez-Júlvez M., Gómez-Moreno C., Schmeits J. L., Tollin G. (1997). Structure-Function
Relationships in Anabaena Ferredoxin: Correlations between X-ray Crystal
Structures, Reduction Potentials, and Rate Constants of Electron Transfer
to Ferredoxin:NADP+ Reductase for Site-Specific Ferredoxin Mutants. Biochemistry.

[11] Vidakovic M., Fraczkiewicz G., Dave B. C., Czernuszewicz R. S., Germanas J. P. (1995). The Environment
of [2Fe-2S] Clusters in Ferredoxins:
The Role of Residue 45 Probed by Site-Directed Mutagenesis. Biochemistry.

[12] Aoki M., Ishimori K., Morishima I., Wada Y. (1998). Roles of valine-98
and glutamic acid-72 of putidaredoxin in the electron-transfer complexes
with NADH-putidaredoxin reductase and P450cam. Inorg. Chim. Acta.

[13] Akashi T., Matsumura T., Taniguchi I., Hase T. (1997). Mutational analysis
of the redox properties of the [2Fe-2S] cluster in plant ferredoxins. J. Inorg. Biochem..

[14] Taniguchi I., Miyahara A., Iwakiri K.-i., Hirakawa Y., Hayashi K., Nishiyama K., Akashi T., Hase T. (1997). Electrochemical Study
of Biological Functions of Particular Evolutionary Conserved Amino
Acid Residues Using Mutated Molecules of Maize Ferredoxin. Chem. Lett..

[15] Akashi T., Matsumura T., Ideguchi T., Iwakiri K. i., Kawakatsu T., Taniguchi I., Hase T. (1999). Comparison of the Electrostatic Binding
Sites on the Surface of Ferredoxin for Two Ferredoxin-dependent Enzymes,
Ferredoxin-NADP+ Reductase and Sulfite Reductase. J. Biol. Chem..

[16] Bellei M., Battistuzzi G., Wu S.-p., Mansy S. S., Cowan J. A., Sola M. (2010). Control of
reduction thermodynamics in [2Fe–2S] ferredoxins:
Entropy–enthalpy compensation and the influence of surface
mutations. J. Inorg. Biochem..

[17] Zöllner A., Hannemann F., Lisurek M., Bernhardt R. (2002). Deletions
in the loop surrounding the iron–sulfur cluster of adrenodoxin
severely affect the interactions with its native redox partners adrenodoxin
reductase and cytochrome P450scc (CYP11A1). J. Inorg. Biochem..

[18] Hannemann F., Rottmann M., Schiffler B., Zapp J., Bernhardt R. (2001). The Loop Region
Covering the Iron-Sulfur Cluster in Bovine Adrenodoxin Comprises a
New Interaction Site for Redox Partners. J.
Biol. Chem..

[19] Beckert V., Bernhardt R. (1997). Specific Aspects
of Electron Transfer from Adrenodoxin
to Cytochromes P450scc and P45011*β*. J. Biol. Chem..

[20] Zuris J. A., Halim D. A., Conlan A. R., Abresch E. C., Nechushtai R., Paddock M. L., Jennings P. A. (2010). Engineering the Redox Potential over
a Wide Range within a New Class of FeS Proteins. J. Am. Chem. Soc..

[21] Baxter E. L., Zuris J. A., Wang C., Vo P. L. T., Axelrod H. L., Cohen A. E., Paddock M. L., Nechushtai R., Onuchic J. N., Jennings P. A. (2013). Allosteric control
in a metalloprotein
dramatically alters function. Proc. Natl. Acad.
Sci. U.S.A..

[22] Guergova-Kuras M., Kuras R., Ugulava N., Hadad I., Crofts A. R. (2000). Specific
Mutagenesis of the Rieske Iron-Sulfur Protein in Rhodobacter sphaeroides
Shows That both the Thermodynamic Gradient and the pK of the Oxidized
Form Determine the Rate of Quinol Oxidation by the bc1 Complex. Biochemistry.

[23] Kolling D. J., Brunzelle J. S., Lhee S., Crofts A. R., Nair S. K. (2007). Atomic
Resolution Structures of Rieske Iron-Sulfur Protein: Role of Hydrogen
Bonds in Tuning the Redox Potential of Iron-Sulfur Clusters. Structure.

[24] Rodríguez-Maciá P., Pawlak K., Rüdiger O., Reijerse E. J., Lubitz W., Birrell J. A. (2017). Intercluster Redox Coupling Influences Protonation
at the H-cluster in [FeFe] Hydrogenases. J.
Am. Chem. Soc..

[25] Dereli B., Baer M. D., Peters J. W., Raugei S. (2025). The Properties That
Allow Tuning the Reduction Potentials over a Volt Range in Biological
Iron/Sulfur Clusters. J. Phys. Chem. Lett..

[26] Sulpizi M., Raugei S., VandeVondele J., Carloni P., Sprik M. (2007). Calculation
of Redox Properties: Understanding Short- and Long-Range Effects in
Rubredoxin. J. Phys. Chem. B.

[27] Langen R., Jensen G., Jacob U., Stephens P., Warshel A. (1992). Protein control
of iron-sulfur cluster redox potentials. J.
Biol. Chem..

[28] Mouesca J.-M., Chen J. L., Noodleman L., Bashford D., Case D. A. (1994). Density
Functional/Poisson-Boltzmann Calculations of Redox Potentials for
Iron-Sulfur Clusters. J. Am. Chem. Soc..

[29] Greco C., Bruschi M., Fantucci P., Ryde U., De Gioia L. (2011). Mechanistic
and Physiological Implications of the Interplay among Iron–Sulfur
Clusters in [FeFe]-Hydrogenases. A QM/MM Perspective. J. Am. Chem. Soc..

[30] Liao R.-Z., Zhang J.-X., Lin Z., Siegbahn P. E. (2021). Antiferromagnetically
coupled [Fe8S9] cluster catalyzed acetylene reduction in a nitrogenase-like
enzyme DCCPCh: Insights from QM/MM calculations. J. Catal..

[31] Sundararajan M., Hillier I. H., Burton N. A. (2006). Structure
and Redox Properties of
the Protein, Rubredoxin, and Its Ligand and Metal Mutants Studied
by Electronic Structure Calculation. J. Phys.
Chem. A.

[32] Sattelle B. M., Sutcliffe M. J. (2008). Calculating
Chemically Accurate Redox Potentials for
Engineered Flavoproteins from Classical Molecular Dynamics Free Energy
Simulations. J. Phys. Chem. A.

[33] van
den Bosch M., Swart M., Snijders† J. G., Berendsen H. J. C., Mark A. E., Oostenbrink C., van Gunsteren W. F., Canters G. W. (2005). Calculation of the Redox Potential
of the Protein Azurin and Some Mutants. ChemBioChem.

[34] Chen C. G., Nardi A. N., Amadei A., D’Abramo M. (2022). Theoretical
Modeling of Redox Potentials of Biomolecules. Molecules.

[35] Silvestri G., Arrigoni F., Persico F., Bertini L., Zampella G., De Gioia L., Vertemara J. (2023). Assessing
the Performance of Non-Equilibrium
Thermodynamic Integration in Flavodoxin Redox Potential Estimation. Molecules.

[36] Jafari S., Tavares Santos Y. A., Bergmann J., Irani M., Ryde U. (2022). Benchmark
Study of Redox Potential Calculations for Iron–Sulfur Clusters
in Proteins. Inorg. Chem..

[37] Galuzzi B. G., Mirarchi A., Viganò E. L., De Gioia L., Damiani C., Arrigoni F. (2022). Machine Learning for
Efficient Prediction of Protein
Redox Potential: The Flavoproteins Case. J.
Chem. Inf. Model..

[200] Franke P., Freiberger S., Zhang L., Einsle O. (2025). Conformational
protection of molybdenum nitrogenase by Shethna protein II. Nature.

[38] Boncella A. E., Sabo E. T., Santore R. M., Carter J., Whalen J., Hudspeth J. D., Morrison C. N. (2022). The expanding utility
of iron-sulfur
clusters: Their functional roles in biology, synthetic small molecules,
maquettes and artificial proteins, biomimetic materials, and therapeutic
strategies. Coord. Chem. Rev..

[39] Zanello P. (2013). The competition
between chemistry and biology in assembling iron–sulfur derivatives.
Molecular structures and electrochemistry. Part I. Fe­(S_γ_Cys)­4 proteins. Coord. Chem. Rev..

[40] Zanello P. (2014). The competition
between chemistry and biology in assembling iron–sulfur derivatives.
Molecular structures and electrochemistry. Part II. [Fe2S2]­(S_γ_Cys)­4 proteins. Coord. Chem. Rev..

[41] Zanello P. (2016). The competition
between chemistry and biology in assembling iron–sulfur derivatives.
Molecular structures and electrochemistry. Part III. [Fe2S2]­(Cys)­3­(X)
(X = Asp, Arg, His) and [Fe2S2]­(Cys)­2­(His)­2 proteins. Coord. Chem. Rev..

[42] Zanello P. (2017). Structure
and electrochemistry of proteins harboring iron-sulfur clusters of
different nuclearities. Part I. [4Fe-4S]+[2Fe-2S] iron-sulfur proteins. J. Struct. Biol..

[43] Zanello P. (2018). Structure
and electrochemistry of proteins harboring iron-sulfur clusters of
different nuclearities. Part III. [4Fe-4S], [3Fe-4S] and [2Fe-2S]
iron-sulfur proteins. J. Struct. Biol..

[44] Lin I.-J., Gebel E. B., Machonkin T. E., Westler W. M., Markley J. L. (2005). Changes
in hydrogen-bond strengths explain reduction potentials in 10 rubredoxin
variants. Proc. Natl. Acad. Sci. U.S.A..

[45] Bak D. W., Elliott S. J. (2014). Alternative FeS cluster ligands:
tuning redox potentials
and chemistry. Curr. Opin. Chem. Biol..

[46] Abriata L. A., Palzkill T., Dal Peraro M. (2015). How Structural
and Physicochemical
Determinants Shape Sequence Constraints in a Functional Enzyme. PLoS One.

[47] Ugulava N. B., Crofts A. R. (1998). CD-monitored redox titration of the Rieske Fe-S protein
of Rhodobacter sphaeroides: pH dependence of the midpoint potential
in isolated bc1 complex and in membranes. FEBS
Lett..

[48] Werth M. T., Cecchini G., Manodori A., Ackrell B. A., Schröder I., Gunsalus R. P., Johnson M. K. (1990). Site-directed
mutagenesis of conserved
cysteine residues in Escherichia coli fumarate reductase: modification
of the spectroscopic and electrochemical properties of the [2Fe-2S]
cluster. Proc. Natl. Acad. Sci. U.S.A..

[49] Lubner C. E. (2017). Mechanistic insights into energy conservation
by flavin-based electron
bifurcation. Nat. Chem. Biol..

[50] Demmer J. K., Rupprecht F. A., Eisinger M. L., Ermler U., Langer J. D. (2016). Ligand
binding and conformational dynamics in a flavin-based electron-bifurcating
enzyme complex revealed by Hydrogen–Deuterium Exchange Mass
Spectrometry. FEBS Lett..

[51] Buckel W., Thauer R. K. (2018). Flavin-Based Electron Bifurcation, A New Mechanism
of Biological Energy Coupling. Chem. Rev..

[52] Min J., Ali F., Brooks B. R., Bruce B. D., Amin M. (2025). Predicting Iron–Sulfur
Cluster Redox Potentials: A Simple Model Derived from Protein Structures. ACS Omega.

[53] Era I., Kitagawa Y., Yasuda N., Kamimura T., Amamizu N., Sato H., Cho K., Okumura M., Nakano M. (2021). Theoretical
Study on Redox Potential Control of Iron-Sulfur Cluster by Hydrogen
Bonds: A Possibility of Redox Potential Programming. Molecules.

[54] Kitagawa Y., Shoji M., Saito T., Nakanishi Y., Koizumi K., Kawakami T., Okumura M., Yamaguchi K. (2008). Theoretical
studies on effects of hydrogen bonds attaching to cysteine ligands
on 4Fe-4S clusters. Int. J. Quantum Chem..

[55] DeRosha D. E., Chilkuri V. G., Van Stappen C., Bill E., Mercado B. Q., DeBeer S., Neese F., Holland P. L. (2019). Planar three-coordinate
iron sulfide in a synthetic [4Fe-3S] cluster with biomimetic reactivity. Nat. Chem..

[56] Shomura Y., Yoon K.-S., Nishihara H., Higuchi Y. (2011). Structural basis for
a [4Fe-3S] cluster in the oxygen-tolerant membrane-bound [NiFe]-hydrogenase. Nature.

[57] Kisgeropoulos E. C., Artz J. H., Blahut M., Peters J. W., King P. W., Mulder D. W. (2024). Properties of the
iron-sulfur cluster electron transfer
relay in an [FeFe]-hydrogenase that is tuned for H2 oxidation catalysis. J. Biol. Chem..

[58] Artz J. H., Mulder D. W., Ratzloff M. W., Lubner C. E., Zadvornyy O. A., LeVan A. X., Williams S. G., Adams M. W. W., Jones A. K., King P. W., Peters J. W. (2017). Reduction
Potentials of [FeFe]-Hydrogenase
Accessory Iron–Sulfur Clusters Provide Insights into the Energetics
of Proton Reduction Catalysis. J. Am. Chem.
Soc..

[59] del
Barrio M., Sensi M., Orain C., Baffert C., Dementin S., Fourmond V., Léger C. (2018). Electrochemical
Investigations of Hydrogenases and Other Enzymes That Produce and
Use Solar Fuels. Acc. Chem. Res..

[60] La
Gatta S., Leone L., Maglio O., De Fenza M., Nastri F., Pavone V., Chino M., Lombardi A. (2021). Unravelling
the Structure of the Tetrahedral Metal-Binding Site in METP3 through
an Experimental and Computational Approach. Molecules.

[61] Waser V., Ward T. R. (2023). Aqueous stability
and redox chemistry of synthetic
[Fe4S4] clusters. Coord. Chem. Rev..

[62] Chino M., Di Costanzo L. F., Leone L., La Gatta S., Famulari A., Chiesa M., Lombardi A., Pavone V. (2023). Designed Rubredoxin
miniature in a fully artificial electron chain triggered by visible
light. Nat. Commun..

[63] Di
Costanzo L. F., Sgueglia G., Orlando C., Polentarutti M., Leone L., La Gatta S., De Fenza M., De Gioia L., Lombardi A., Arrigoni F., Chino M. (2025). Structural insights
into temperature-dependent dynamics of METPsc1, a miniaturized electron-transfer
protein. J. Inorg. Biochem..

[64] Hosseinzadeh P., Lu Y. (2016). Design and fine-tuning redox potentials of metalloproteins involved
in electron transfer in bioenergetics. Biochimica
et Biophysica Acta (BBA) - Bioenergetics.

[65] Beard H., Cholleti A., Pearlman D., Sherman W., Loving K. A. (2013). Applying
Physics-Based Scoring to Calculate Free Energies of Binding for Single
Amino Acid Mutations in Protein-Protein Complexes. PLoS One.

[66] Schrödinger Release 2025–2; Maestro, Schrödinger, LLC, New York, NY, 2025.

[67] Schymkowitz J., Borg J., Stricher F., Nys R., Rousseau F., Serrano L. (2005). The FoldX web server: an online force
field. Nucleic Acids Res..

[68] Montanucci L., Capriotti E., Frank Y., Ben-Tal N., Fariselli P. (2019). DDGun: an
untrained method for the prediction of protein stability changes upon
single and multiple point variations. BMC Bioinf..

[69] Rodrigues C. H., Pires D. E., Ascher D. B. (2018). DynaMut: predicting
the impact of
mutations on protein conformation, flexibility and stability. Nucleic Acids Res..

[70] Friedman J. H. (2001). Greedy
function approximation: A gradient boosting machine. Ann. Stat.

[71] Chen, T. ; Guestrin, C. XGBoost: A Scalable Tree Boosting System. Proceedings of the 22nd ACM SIGKDD International Conference on Knowledge Discovery and Data Mining 2016, 785–794.

