# Supplementary material for: Predicting Metalloprotein Redox Potentials with Machine Learning: A Focus on Iron–Sulfur Systems
Source: J Chem Inf Model. 2025 Oct 30;65(21):11631–43. doi: 10.1021/acs.jcim.5c01752 (PMC12606649; doi:10.1021/acs.jcim.5c01752)
Supplement: Supplementary file 1 [file ci5c01752_si_001.pdf]

Predicting metalloprotein redox potentials with Machine Learning: a focus on iron-sulfur systems

Francesca Persico,[†,#] Bruno G. Galuzzi,[‡,¶,#] Miriana Pellegrino,[§] Anne-Lise Claudel,[‖] Luca De Gioia,[†] Flavia Nastri,[§] Gianfranco Gilardi,[‖] Chiara Damiani,[†,⊥] Francesca Valetti,[‖] Marco Chino,[§] and Federica Arrigoni[*,†]

†*Department of Biotechnology and Biosciences, University of Milano-Bicocca, Piazza dell'Ateneo Nuovo, 1 - 20126, Milan, Italy.*

‡*Institute of Bioimaging and Complex Biological Systems, National Research Council, Via Fratelli Cervi, 96 - 20054, Segrate, Italy.*

¶*National Biodiversity Future Center, Piazza Marina, 61 - 90133, Palermo, Italy*

§*Department of Chemical Sciences, University of Naples "Federico II", Via Cinthia, 26 - 80126, Napoli, Italy.*

‖*Department of Life Sciences and Systems Biology, University of Torino, Via Verdi, 8 - 10124, Torino, Italy.*

⊥*Laboratorio InfoLife, Consorzio Interuniversitario Nazionale per l'Informatica, Via Ariosto, 25 - 00185, Roma, Italy.*

#*Francesca Persico and Bruno Giovanni Galuzzi equally contributed to this work.*

E-mail: federica.arrigoni@unimib.it

Additional training performance

Table S1 shows the performance metrics of the best model for each type of training. Introducing the experimental methodology as a categorical feature to train the model doesn't improve the accuracy and doubles the time of execution. The "no mutants" training is carried on excluding all *in silico*-generated mutants. Further details on these models training performance can be found in supporting file S2.

Table S1: Performance metrics(Mean Absolute Error (MAE), Root Mean Squared Error (RMSE), R2, SC, and execution time) for different Machine Learning (ML) models.

Model	MAE (mV)	RMSE (mV)	R^2	SC	Time of execution (h:min:s)
**A-**XGB	39.9 ± 4.2	57.7 ± 8.4	0.94 ± 0.02	0.97 ± 0.01	22:38:53
**B-**XGB	40.5 ± 4.7	60.1 ± 11.3	0.94 ± 0.03	0.95 ± 0.02	2:44:57
**Methodologies**	38.2 ± 3.9	56.5 ± 8.4	0.94 ± 0.02	0.95 ± 0.02	45:37:37
**No mutants**	53.9 ± 13.9	80.2 ± 22.7	0.89 ± 0.08	0.88 ± 0.07	9:51:7

Additional data

Here we report:

- information on structural resolution of the dataset entries;
- prediction results of test proteins excluded from the training dataset;
- SHAP analysis results for the different models trained;
- $\Delta\Delta$G of *in silico*-generated mutants.

In Figures S4, S5, and S12 the plots represent:

(a) SHAP summary plot. The x-axis represents the means of the absolute value of the SHAP value, which represents the impact on model output of that particular molecular descriptor.

(b) SHAP violin plot. The x-axis represents the impact of the molecular descriptor on the predicted Reduction Potential (RP), the color represents the value of the molecular descriptor. Negative SHAP values represent a negative correlation between the molecular descriptor and the RP.

List of Figures

S1 PDB resolution distribution and correlation with errors. 4

S2 Test results on randomly excluded proteins. 4

S3 Test results on two excluded mutants for ferredoxin 1AYF, Rieske 1ZRT, mitoNEET 2QH7 and rubredoxin 1BFY 5

S4 A-XGB model $r_1 = 11$, $r_2 = 4$ 6

S5 B-XGB model 7

S6 Mann–Whitney U rank test for different training 8

S7 No Long-Range models 9

S8 No Medium-Range models 10

S9 No Short-Range models 11

S10 Short-Range Only models 12

S11 Medium-Range Only models 13

S12 Long-Range Only model 14

S13 ΔΔG values of 1FXA mutants 15

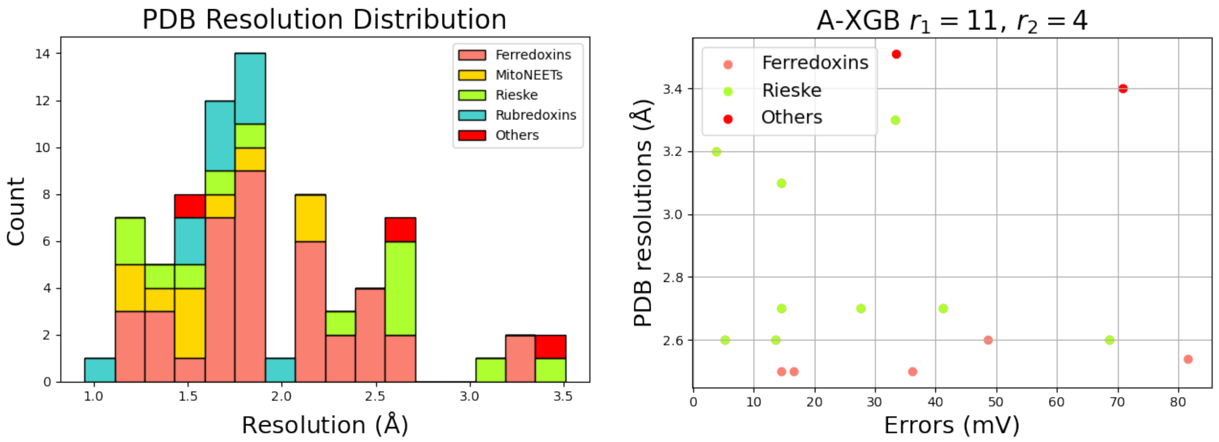

(a). Resolution distribution of dataset entries. **(b).** Correlation between errors and PDB resolution for proteins with resolution over 2.5 Å.

Figure S1: PDB resolution distribution and correlation with errors.

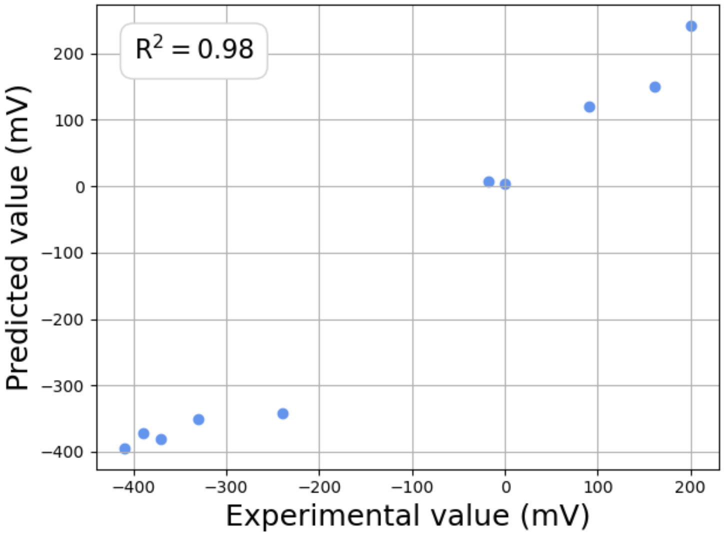

Figure S2: Test results on randomly excluded proteins.

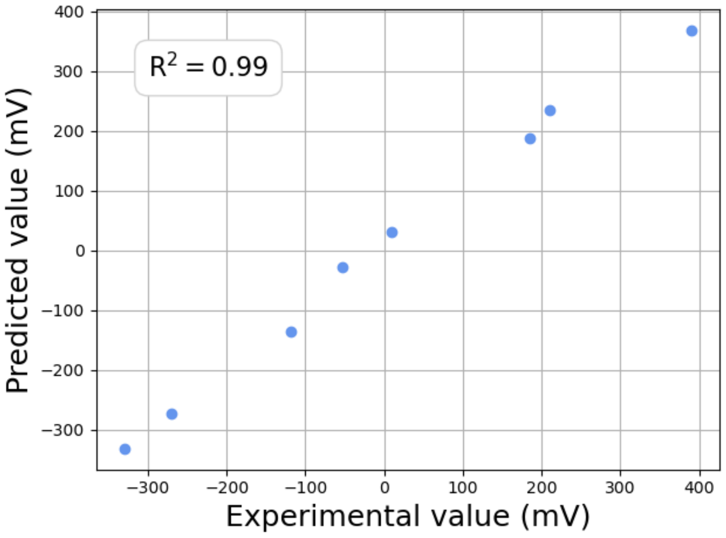

(a). Correlation between experimental and predicted redox potential of protein excluded from training dataset.

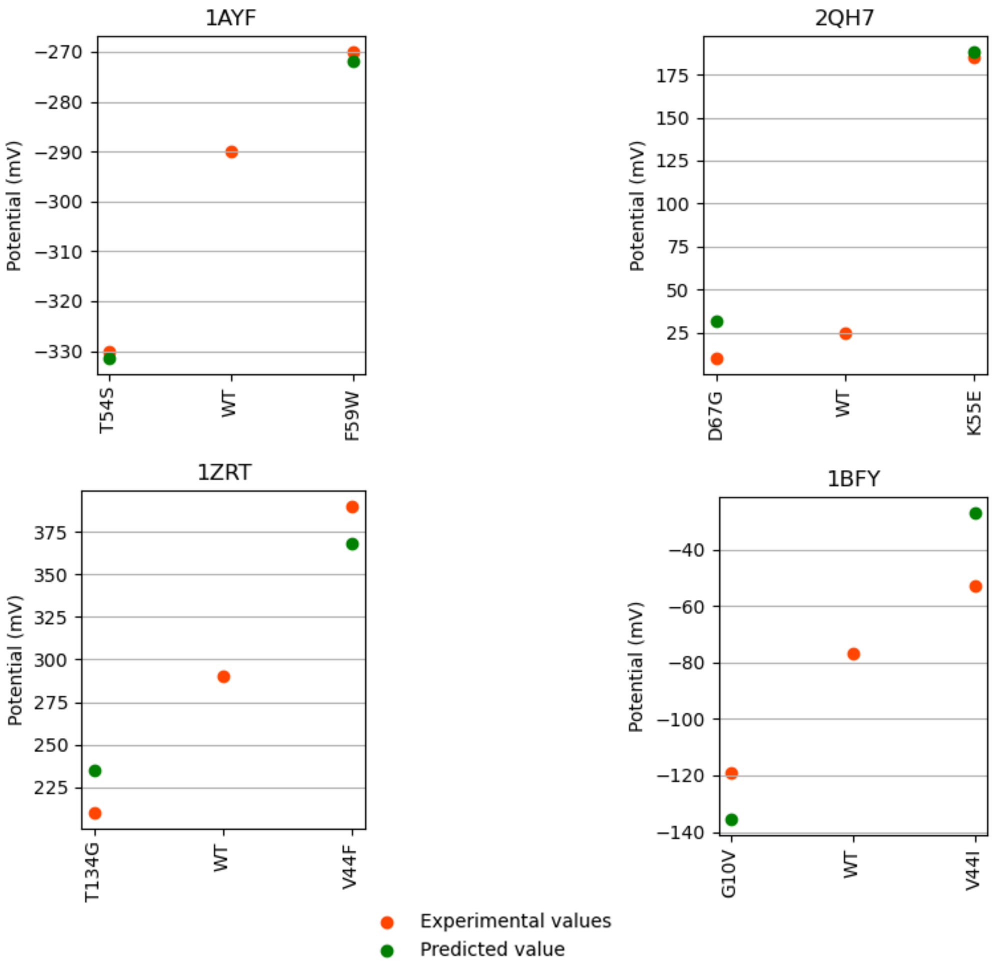

(b). Predicted and experimental redox potential of test proteins VS experimental redox potential of wild type protein.

Figure S3: Test results on two excluded mutants for ferredoxin 1AYF, Rieske 1ZRT, mitoNEET 2QH7 and rubredoxin 1BFY

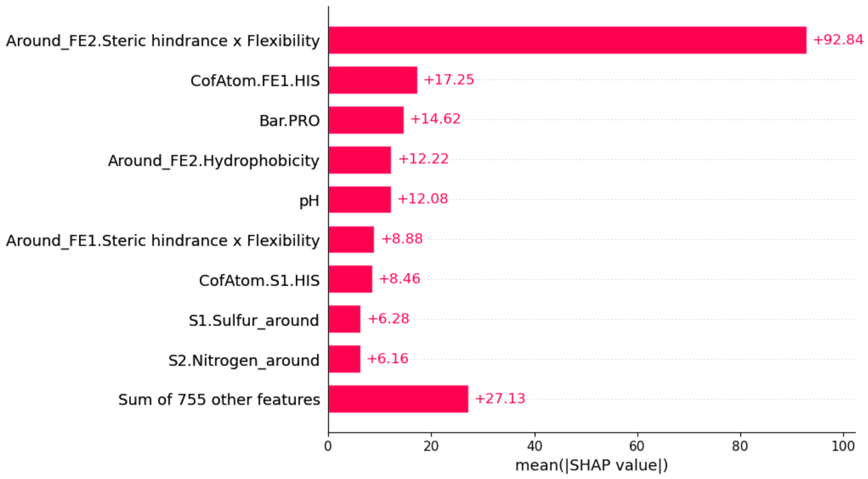

(a)

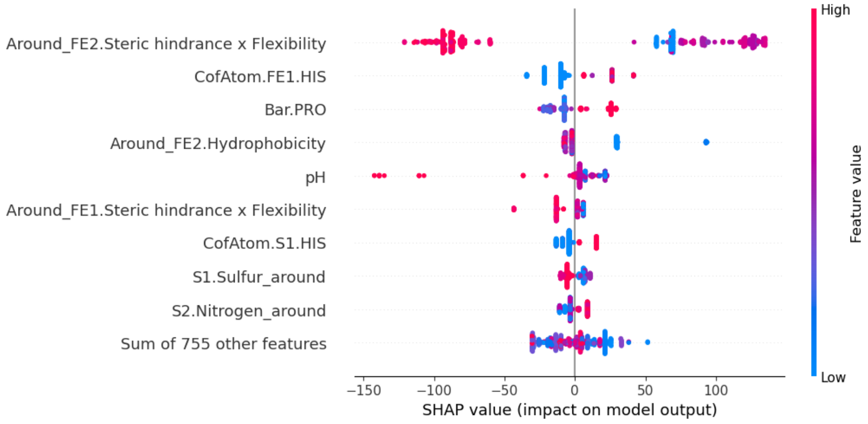

(b)

Figure S4: A-XGB model $r_1 = 11$, $r_2 = 4$

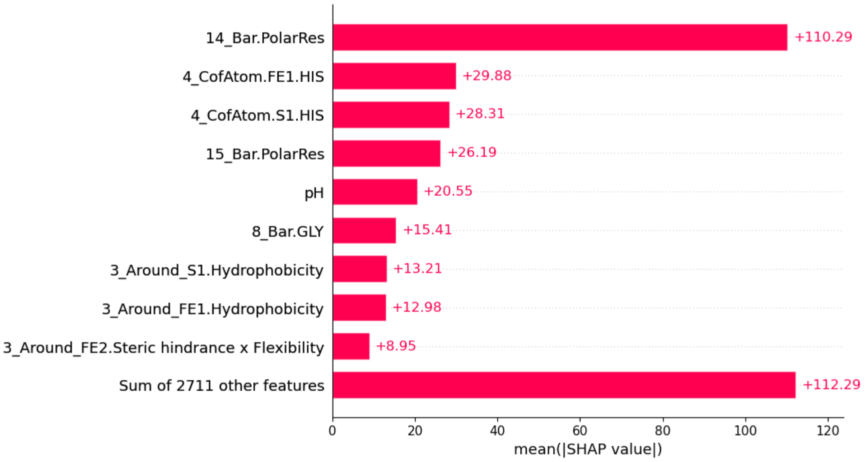

(a)

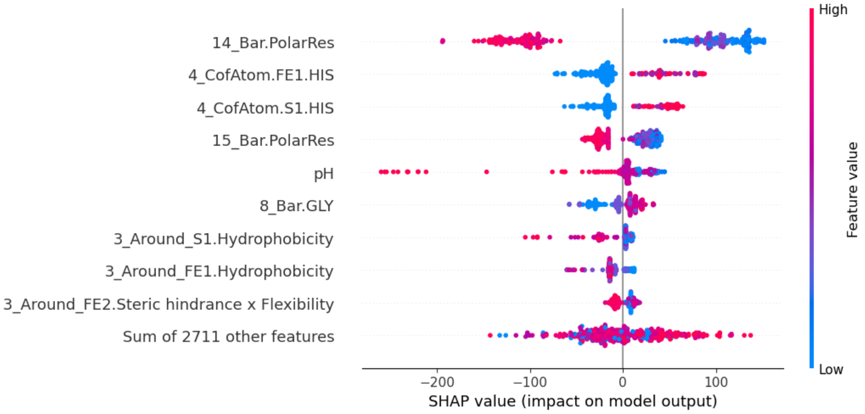

(b)

Figure S5: B-XGB model

This model is trained with 2721 molecular descriptors, which are the same descriptors employed in A-XGB models, merged together without duplications.

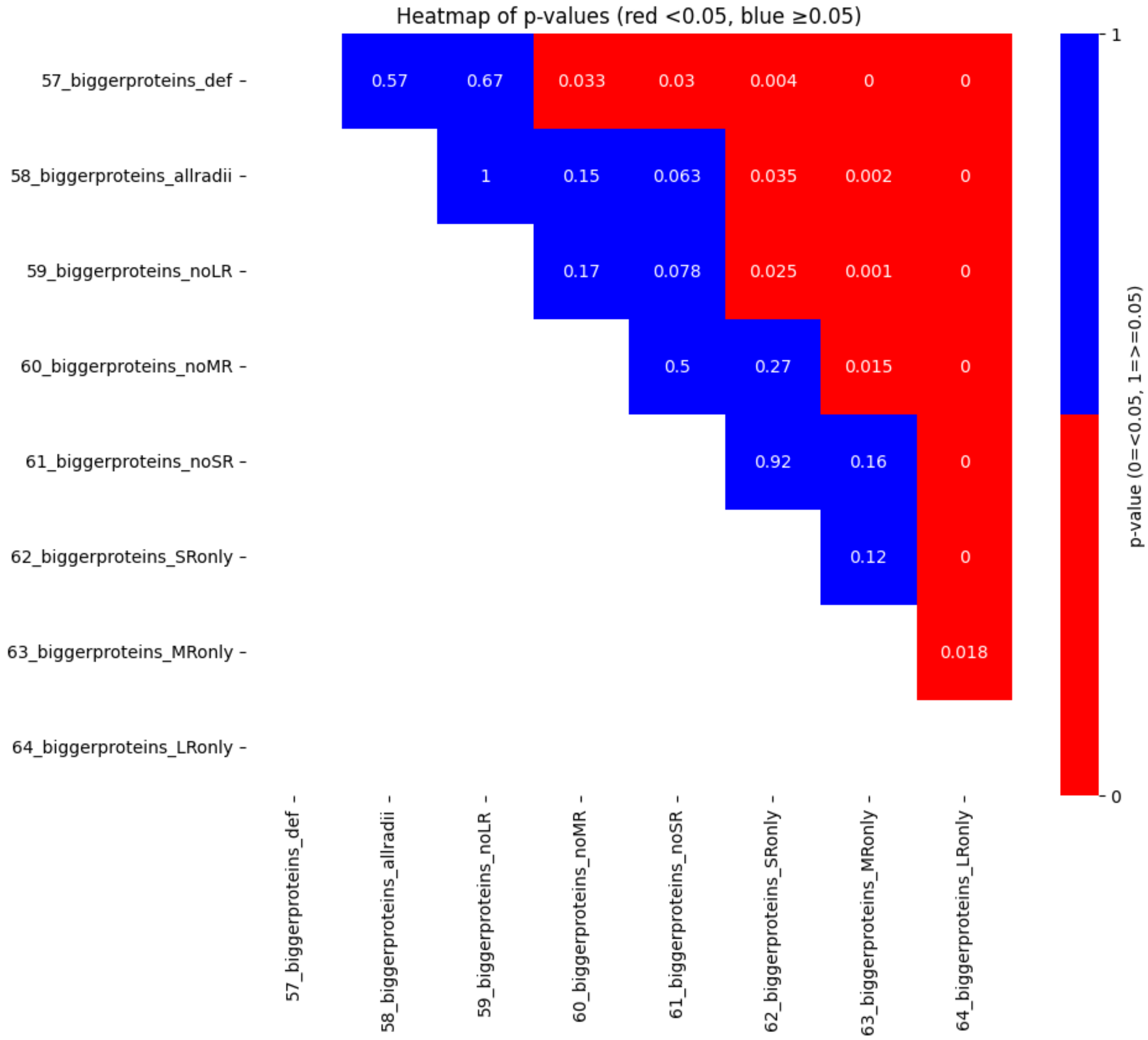

Figure S6: Mann–Whitney U rank test for different training

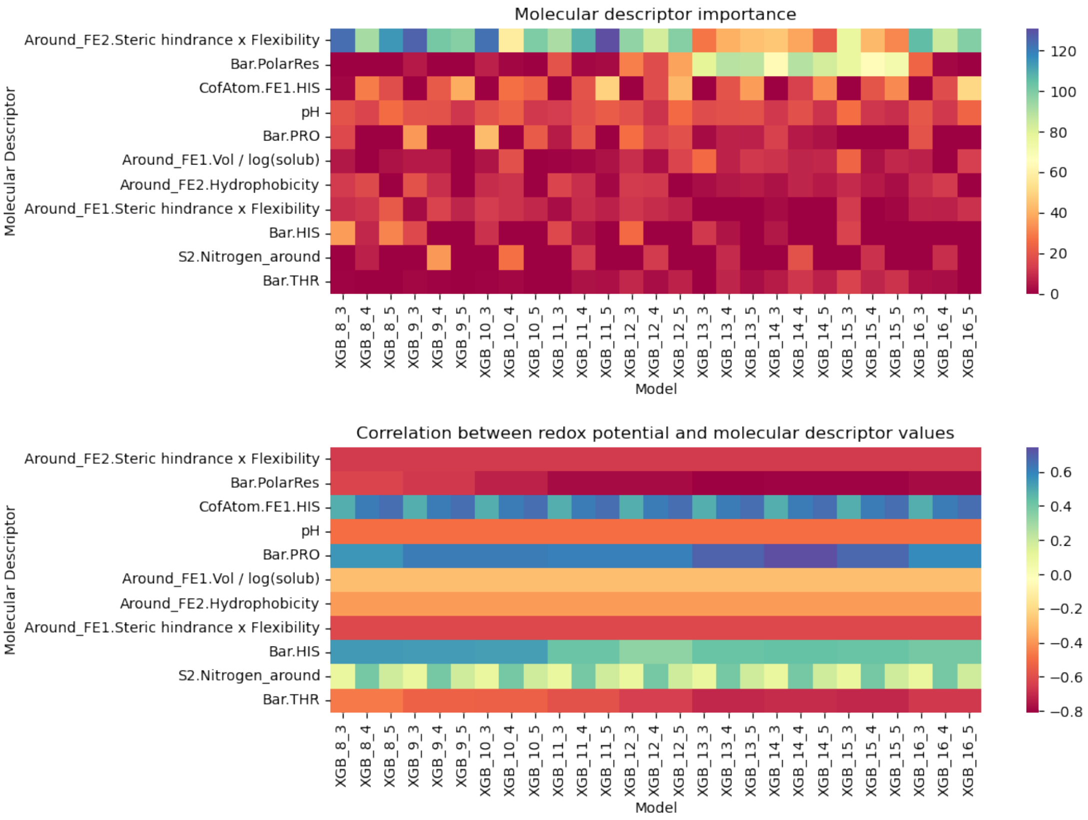

Figure S7: No Long-Range models

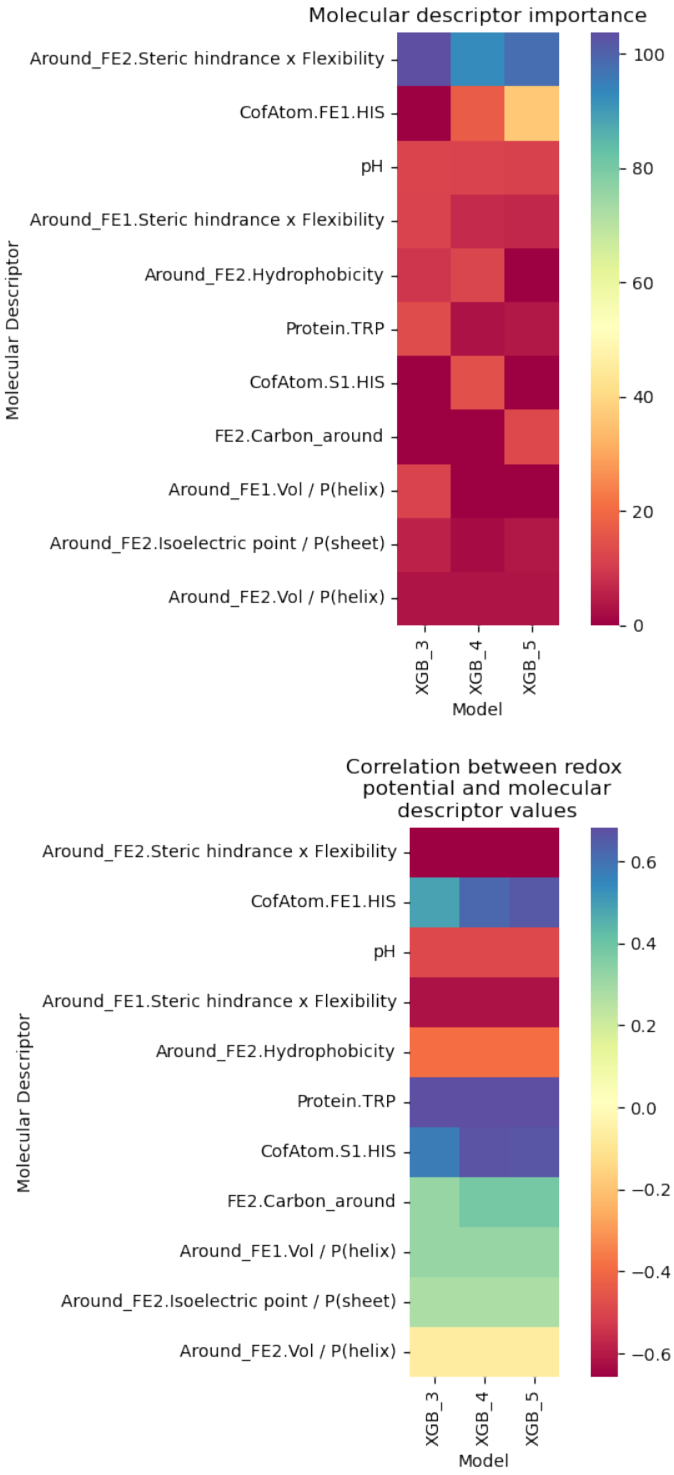

Figure S8: No Medium-Range models

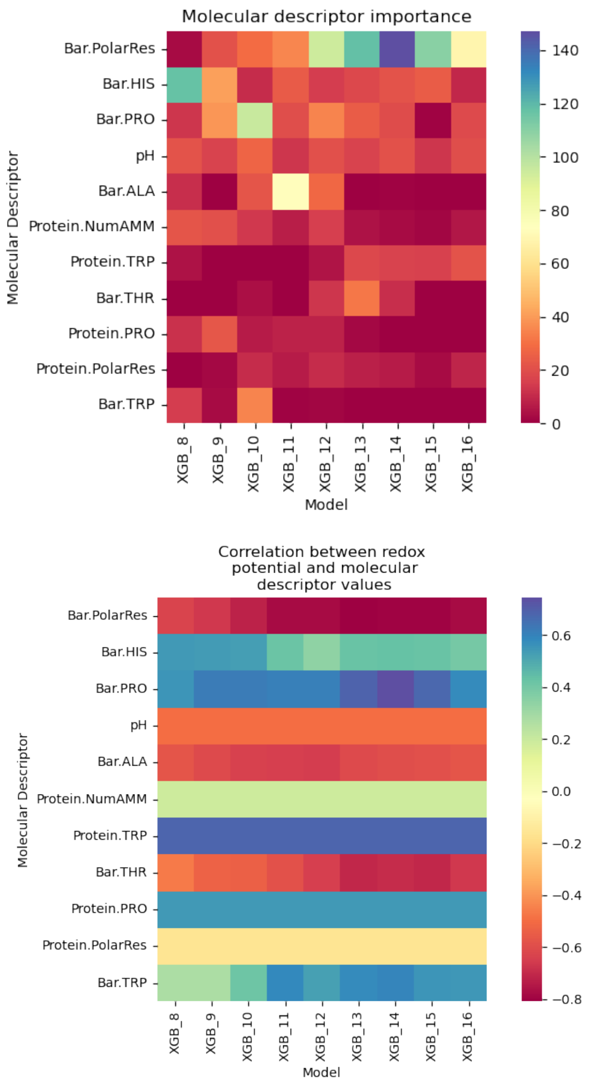

Figure S9: No Short-Range models

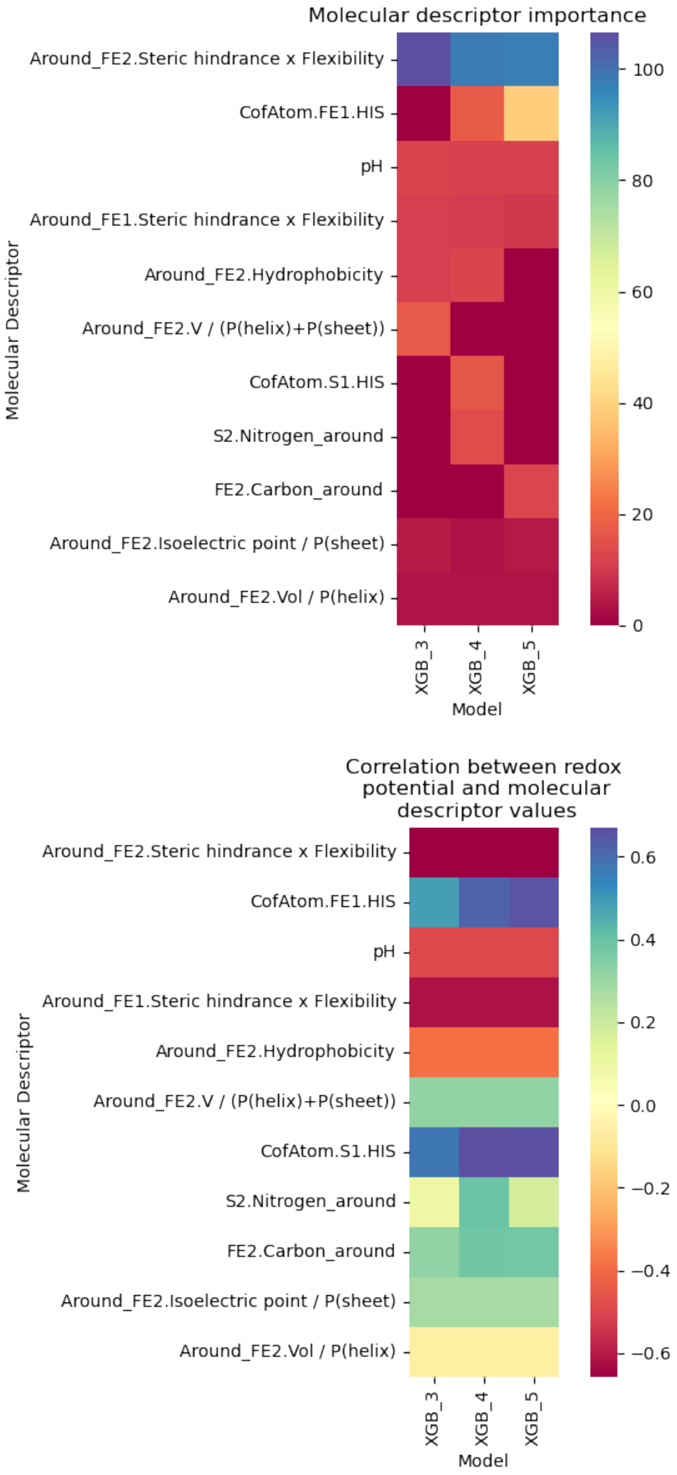

Figure S10: Short-Range Only models

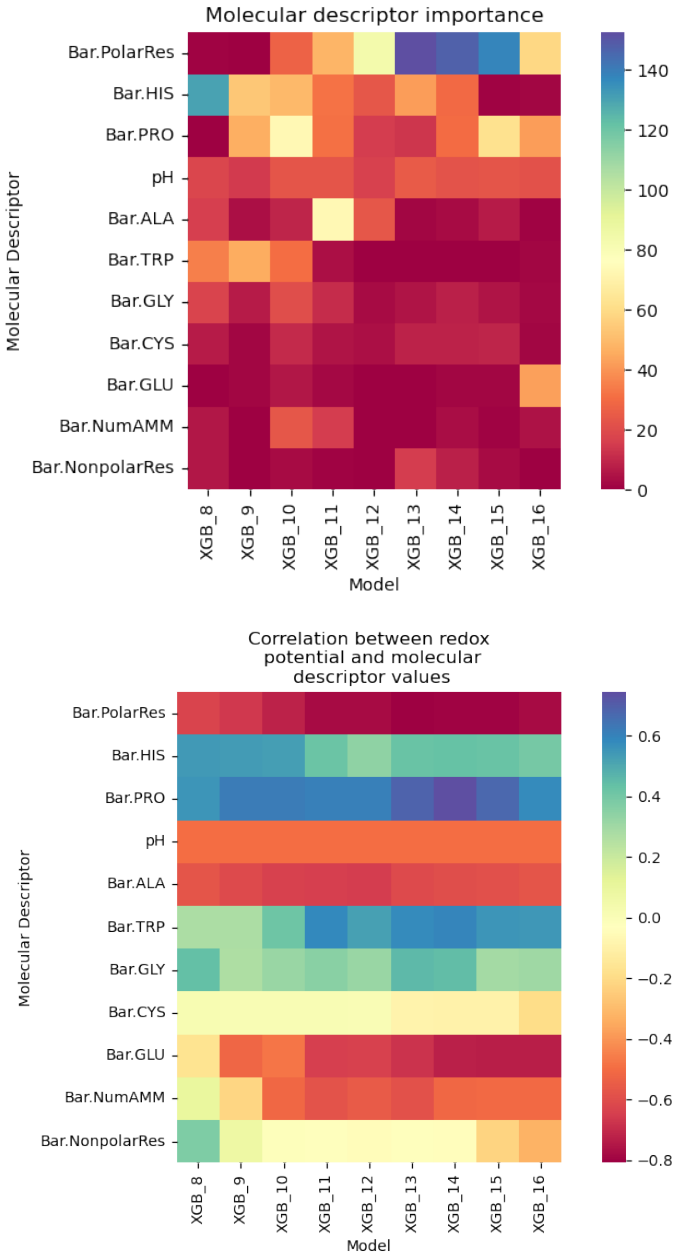

Figure S11: Medium-Range Only models

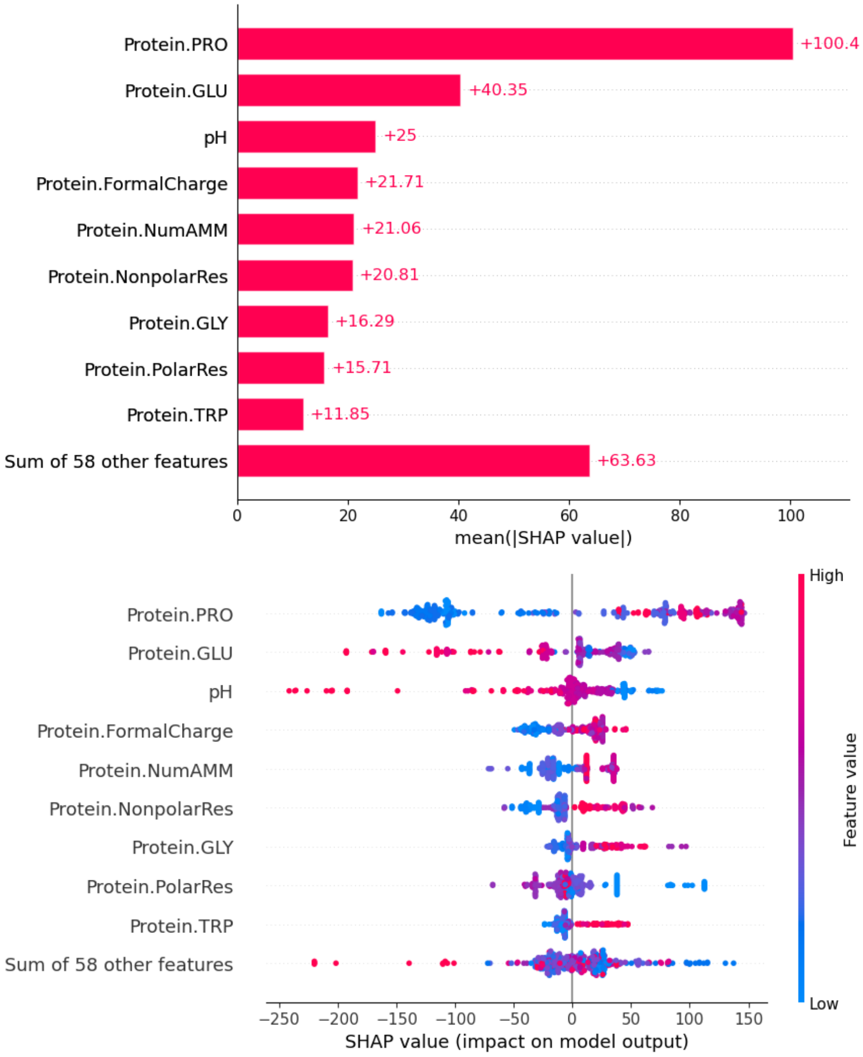

Figure S12: Long-Range Only model

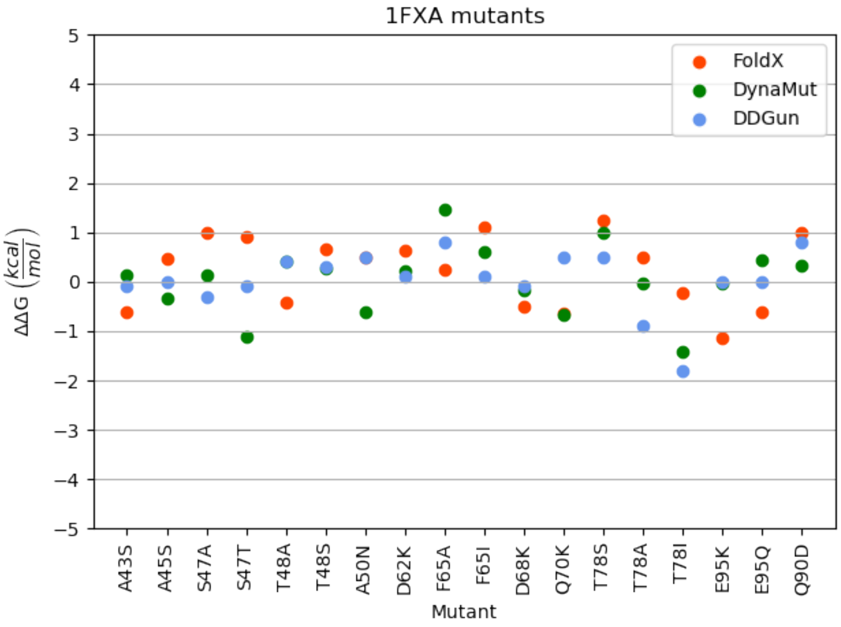

Figure S13: ΔΔG values of 1FXA mutants

The folding ΔΔG values are calculated as $\Delta G_{\mathrm{mutant}} - \Delta G_{\mathrm{wildtype}}$. Thus, positive values indicate that the mutant is less stable than the wild type, and vice versa, across all three predictors.
